# Optimized Genome‐Wide Profiling of Centromeric Chromatin Complexes by ChIP‐seq

**DOI:** 10.1002/cpz1.70471

**Published:** 2026-10-07

**Authors:** Alessia Daponte, Rachel Schwope, Caterina Manzari, Lucia Trisolini, Francesca Antonacci, Michele Morgante, Mario Ventura, Glennis A. Logsdon, Claudia R. Catacchio

**Affiliations:** ^1^ Department of Biosciences, Biotechnology and Environment University of Bari Bari Italy; ^2^ Istituto di Genomica Applicata Udine Italy; ^3^ Department of Agricultural, Food, Environmental and Animal Sciences University of Udine Udine Italy; ^4^ Institute of Biomembranes, Bioenergetics and Molecular Biotechnologies National Research Council Bari Italy; ^5^ Department of Genetics, Epigenetics Institute, Perelman School of Medicine University of Pennsylvania Philadelphia Pennsylvania

**Keywords:** CENP‐A, CENP‐C, centromere, centromeric chromatin, ChIP‐seq, chromatin immunoprecipitation, repetitive DNA

## Abstract

Centromeres are specialized chromosomal regions essential for faithful chromosome segregation and genome stability. Their highly repetitive DNA composition and epigenetically defined chromatin state present significant challenges for genome‐wide profiling approaches such as chromatin immunoprecipitation followed by sequencing (ChIP‐seq). Standard ChIP‐seq workflows often perform poorly at centromeric regions, resulting in low signal‐to‐noise ratios and limited reproducibility. This article describes a ChIP‐seq protocol optimized to enrich for and analyze centromeric chromatin from cell lines. The procedure builds on established ChIP‐seq methodologies while incorporating centromere‐focused modifications in nuclei isolation, chromatin fragmentation, immunoprecipitation, library preparation, and data analysis. Detailed step‐by‐step instructions, critical parameters, recommended controls, and troubleshooting guidance are provided to support reproducible implementation. When applied carefully, this protocol enables reliable profiling of centromere‐associated chromatin and can be integrated into broader studies of chromosome organization, centromere function, and genome stability. © 2026 The Author(s). *Current Protocols* published by Wiley Periodicals LLC.

**Basic Protocol 1**: Sucrose gradient–based isolation of nuclei

**Support Protocol 1**: Titration of micrococcal nuclease (MNase)

**Basic Protocol 2**: MNase digestion and immunoprecipitation of CENP‐A chromatin

**Alternate Protocol 1**: Chromatin sonication and immunoprecipitation of CENP‐C chromatin complexes

**Support Protocol 2**: NET blocking and washing of Protein G Sepharose beads

**Basic Protocol 3**: DNA extraction

**Alternate Protocol 2**: Reversal of cross‐links and DNA purification

**Support Protocol 3**: Bead clean‐up

**Basic Protocol 4**: Library preparation and size selection

**Support Protocol 4**: qPCR to assess α‐satellite DNA enrichment

**Basic Protocol 5**: ChIP‐seq data processing and analysis

## INTRODUCTION

Centromeres are specialized chromosomal domains that ensure accurate chromosome segregation during cell division. They provide the foundation for kinetochore assembly, enabling spindle microtubules to attach to chromosomes and ensuring that sister chromatids (or homologous chromosomes in meiosis) are correctly separated to daughter cells. When centromere function is compromised, the risk for aneuploidy and genome instability increases (Nagaoka et al., 2012; Yilmaz et al., 2021). In most eukaryotes, centromeres are primarily defined epigenetically rather than by a unique DNA sequence and are characterized by long tracts of tandem repeats that form satellite DNA arrays. At centromeres, we find a specialized chromatin marked by the histone H3 variant CENP‐A. CENP‐A nucleosomes recruit a network of constitutive centromere proteins, including CENP‐C, that stabilize centromeric chromatin, promote kinetochore formation, and coordinate the timing of centromere assembly with the cell cycle (McKinley & Cheeseman, 2016). Understanding the composition and regulation of centromeric chromatin is therefore critical not only for dissecting the molecular basis of faithful chromosome inheritance, but also for broader questions in genome organization, the evolution of repetitive DNA, and mechanisms that safeguard genome stability.

Several experimental strategies have been proposed to study centromeric chromatin, including immunofluorescence assays, chromatin immunoprecipitation coupled with quantitative polymerase chain reaction (ChIP‐qPCR) using locus‐specific primers, and sequencing‐based methods combined with specialized mapping strategies (Gao et al., 2026; Logsdon et al., 2021; 2024; Logsdon & Eichler, 2022; Niikura & Kitagawa, 2016). Although these methods have provided important insights into centromere composition and organization, each has inherent limitations. Immunofluorescence offers spatial resolution but is largely qualitative and lacks genome‐wide coverage, while ChIP‐qPCR is restricted to a limited number of predefined loci and depends heavily on primer design and repeat annotation. Sequencing‐based approaches can, in principle, achieve broader coverage, but their performance at centromeres is often constrained by technical and analytical challenges (Miga, 2017).

Chromatin immunoprecipitation followed by sequencing (ChIP‐seq) is widely used for genome‐wide profiling of protein–DNA interactions and histone modifications. However, standard ChIP‐seq protocols are poorly suited for centromeric regions due to the highly repetitive nature of centromeric DNA, low mappability of short sequencing reads, and often weak or variable enrichment signals. These challenges can lead to extensive read loss or ambiguous alignments, and to broad and modest enrichment signals rather than sharp, high‐amplitude peaks typical of transcription factors or active chromatin marks, resulting in low signal‐to‐noise ratios, poor reproducibility, and ambiguous interpretation when conventional protocols are applied without modification. As a result, there remains a clear need for practical ChIP‐seq protocols specifically optimized for centromeric chromatin and compatible with commonly used molecular biology workflows and sequencing platforms.

This article describes an optimized ChIP‐seq workflow for the enrichment and analysis of centromeric chromatin (Fig. 1). The workflow can be performed under either native conditions, which are appropriate for histone‐associated centromeric proteins such as CENP‐A (Altemose et al., 2022; Hasson et al., 2013; Kumara Mastrorosa et al., 2024; Logsdon et al., 2021), or cross‐linked conditions, which are recommended for non‐histone centromeric proteins such as CENP‐C that require stabilization of protein–DNA interactions prior to immunoprecipitation (Alonso et al., 2007; Tolomeo et al., 2017; Trazzi et al., 2009). In this workflow, formaldehyde cross‐linking is used to preserve protein–DNA interactions prior to chromatin fragmentation by sonication, followed by reversal of cross‐links during DNA recovery. The procedure builds on established ChIP‐seq methodologies but incorporates specific modifications to improve performance in satellite‐rich genomic regions.

**Figure 1:**
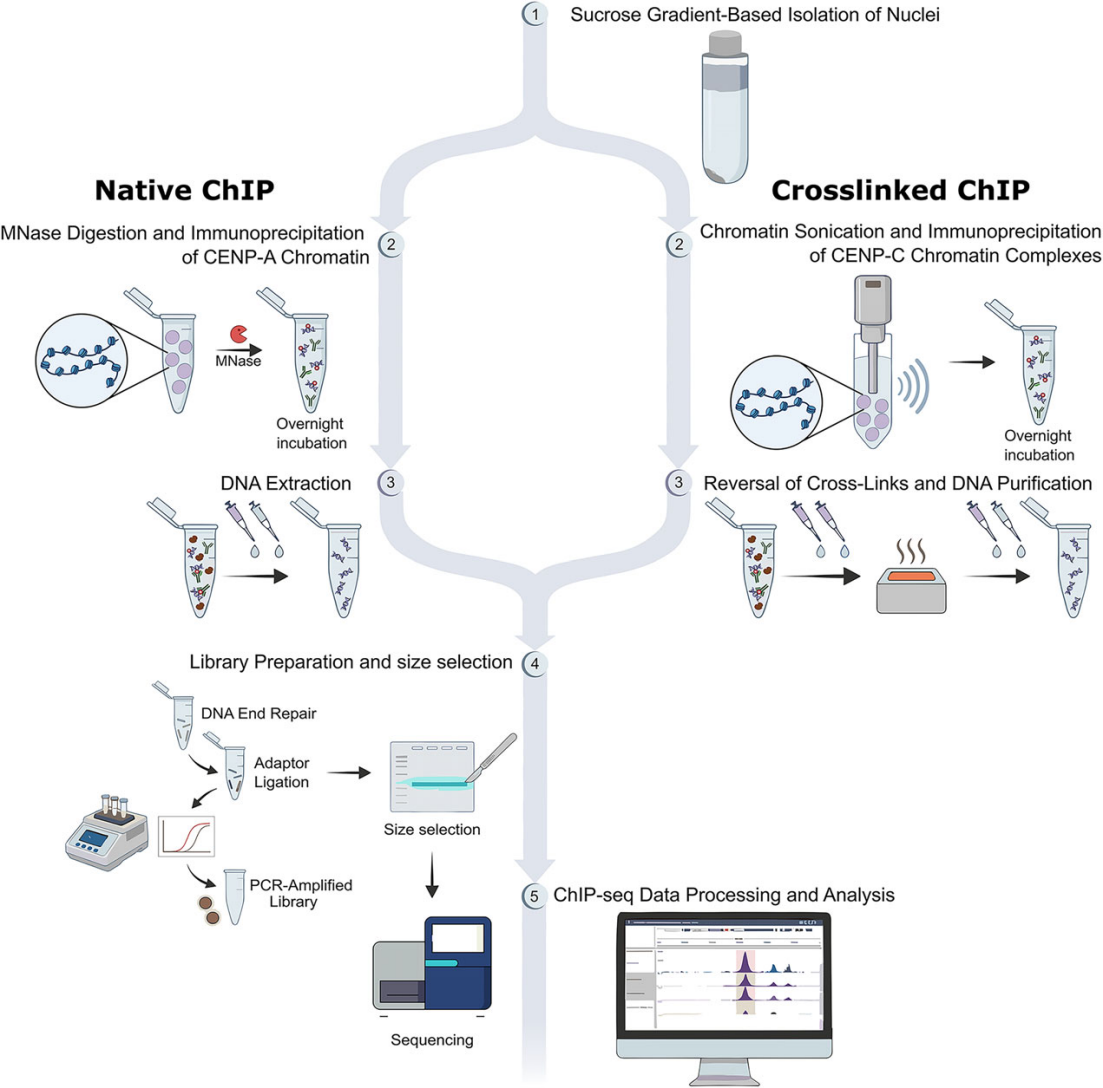
Overview of the centromeric ChIP‐seq protocol described in this article. The workflow begins with sucrose gradient–based isolation of nuclei (Basic Protocol 1) and then branches into two approaches: native ChIP for histone‐associated targets such as CENP‐A, including MNase digestion, immunoprecipitation, and DNA extraction (Basic Protocols 2 to 3), and cross‐linked ChIP for non‐histone proteins such as CENP‐C, including chromatin sonication, immunoprecipitation, cross‐link reversal, and DNA purification (Alternate Protocols 1 to 2). Both workflows converge in library preparation and size selection (Basic Protocol 4) followed by ChIP‐seq data processing and analysis (Basic Protocol 5).

Basic Protocol 1 describes the isolation of intact nuclei from cultured cells by sequential detergent lysis and sucrose gradient centrifugation and serves as the common starting point for both workflows. For native ChIP‐seq applications, Support Protocol 1 describes optimization of micrococcal nuclease (MNase) digestion conditions, followed by Basic Protocol 2, which details MNase digestion and immunoprecipitation of CENP‐A chromatin under native conditions. Alternate Protocol 1 describes the corresponding workflow for cross‐linked samples, including controlled chromatin fragmentation by sonication and immunoprecipitation of centromeric protein–DNA complexes using specific antibodies. Support Protocol 2 describes NET blocking and washing of Protein G Sepharose beads for use in native immunoprecipitation. Basic Protocol 3 outlines DNA extraction from native chromatin preparations, whereas Alternate Protocol 2 describes recovery of immunoprecipitated DNA from cross‐linked samples through protein G bead‐based capture, washing, reversal of cross‐links, and purification. Support Protocol 3 provides instructions for paramagnetic bead‐based DNA clean‐up to remove reaction components while retaining appropriately sized fragments and is applicable to both workflows. Basic Protocol 4 describes low‐input library preparation from ChIP DNA and quantitative size selection optimized for repetitive genomic regions, and Support Protocol 4 presents a quantitative PCR assay to assess enrichment of α‐satellite DNA relative to input controls. Finally, Basic Protocol 5 describes computational processing and analysis of sequencing data, including alignment, normalization, and visualization strategies tailored to centromeric regions.

## STRATEGIC PLANNING

Profiling centromeric chromatin by ChIP‐seq requires attention to several experimental parameters that influence enrichment efficiency and downstream data interpretability. A key feature of this protocol is that each major step has been optimized for the biochemical and computational challenges posed by centromeric chromatin, including repetitive DNA composition, broad enrichment profiles, and reduced read mappability.

This procedure is optimized for cultured cells but can be adapted to primary cells or tissues with appropriate optimization. Because centromeric chromatin represents only a small fraction of total chromatin, sufficient starting material is essential for reproducible enrichment. As a general guideline, at least 2 × 10⁷ cells per immunoprecipitation are recommended when targeting centromere‐associated proteins.

Antibodies should be validated for ChIP applications and, where possible, independently assessed for specificity (e.g., by immunofluorescence). An input DNA control should be included for each sample to enable normalization and enrichment analysis of immunoprecipitated data. An IgG immunoprecipitation control may also be included to evaluate nonspecific background signals.

A key initial decision is whether the target protein should be analyzed under native or cross‐linked conditions. Native ChIP‐seq is generally recommended for histone‐associated centromeric proteins such as CENP‐A, which are stably incorporated into nucleosomes and remain tightly associated with DNA. In contrast, cross‐linked ChIP‐seq is recommended for non‐histone centromeric proteins such as CENP‐C, whose association with chromatin may require stabilization by formaldehyde fixation prior to immunoprecipitation. Consistent chromatin fragmentation is critical for both immunoprecipitation efficiency and downstream analysis and must be adapted to the chosen workflow. For native ChIP‐seq, controlled MNase digestion is critical to obtain predominantly mononucleosomal fragments while preserving centromeric nucleosome integrity; therefore, MNase titration should be performed before processing experimental samples (Support Protocol 1). For cross‐linked samples, sonication conditions must be carefully optimized to achieve reproducible fragment sizes suitable for immunoprecipitation and library preparation (see Critical Parameters in the Commentary section). Over‐fragmentation can reduce recovery of protein–DNA complexes, whereas under‐fragmentation can limit resolution and library complexity. Fragment size distributions should therefore be assessed for each sample (see Commentary) to ensure the suitability of sonication conditions.

The choice of bead system for the immunoprecipitation depends on the workflow. In the native CENP‐A protocol, Protein G Sepharose beads provide efficient recovery of intact nucleosomal complexes during extended incubation and gentle washing steps. In contrast, for cross‐linked CENP‐C samples, Dynabeads Protein G are preferred because magnetic separation accelerates chromatin capture, improves reproducibility during repeated washes, and reduces sample loss during decrosslinking and DNA recovery.

Prior to sequencing, immunoprecipitated DNA should be evaluated by qPCR using primers specific to centromeric sequences and non‐centromeric control loci to verify enrichment relative to input DNA, as described in Support Protocol 4 and the Commentary.

Paired‐end sequencing (e.g., 2 × 150 bp or up to 2 × 250 bp) is recommended to improve alignment and read assignment from repetitive regions. Approximately 10 million reads per antibody are typically sufficient for analysis. When available, ChIP‐seq reads should be mapped to a high‐quality genome assembly generated from the same individual used for the experiment and containing gapless centromeric or satellite sequence annotations. In the absence of such an assembly, the highest‐quality reference genome available for the same species is recommended.

*NOTE*: All protocols involving animals must be reviewed and approved by the appropriate Animal Care and Use Committee and must follow regulations for the care and use of laboratory animals. Appropriate informed consent is necessary for obtaining and use of human study material.

## Basic Protocol 1 SUCROSE GRADIENT–BASED ISOLATION OF NUCLEI

This protocol describes the isolation and purification of intact nuclei from cultured cells using sequential detergent‐based cell lysis followed by sucrose gradient centrifugation and is optimized for the analysis of centromeric DNA–protein interactions. Sequential lysis with mild detergents efficiently disrupts the plasma membrane while preserving nuclear integrity and maintaining stable protein–DNA complexes within highly repetitive centromeric chromatin. Subsequent density‐based separation through a sucrose density gradient removes cytoplasmic components, cellular debris, and partially disrupted material, yielding a highly enriched preparation of intact nuclei suitable for downstream ChIP‐seq applications. This protocol can be performed under either native or cross‐linked conditions, depending on the centromeric target of interest. For histone‐associated proteins, such as CENP‐A, nuclei are typically isolated under native conditions. For non‐histone centromeric proteins, such as CENP‐C and other kinetochore‐associated components, formaldehyde cross‐linking is recommended prior to nuclei isolation to stabilize protein–DNA interactions and preserve chromatin‐associated complexes during downstream processing. When required, cross‐linking should be performed as described in “Cell harvest for cross‐linked ChIP‐seq (CENP‐C) and cross‐linking”.

### Materials

Cells
1× phosphate‐buffered saline (PBS), ice‐cold
Formaldehyde (Sigma‐Aldrich, cat. no. F8775)
Glycine (Sigma‐Aldrich, cat. no. 50046)
Trypan blue (Sigma‐Aldrich, cat. no. T8154)
Buffer I, ice‐cold (see recipe)
Buffer II, ice‐cold (see recipe)
Buffer III, ice‐cold (see recipe)
1 M DTT (Thermo Fisher Scientific, cat. no. R0861)
100 mM PMSF (Thermo Fisher Scientific, cat. no. 36978)
100× Halt protease inhibitor (PI) cocktail (Thermo Fisher Scientific, cat. no. 87786)
Buffer IV, ice‐cold (see recipe)

Refrigerated swinging‐bucket centrifuge (Sorvall ST4 Plus, Thermo Fisher Scientific, cat. no. 75009525)
10‐ml pipettes and pipette aid
Ice bucket and ice
50‐ml tubes (Corning, cat. no. 352070)
Pipettes and low‐retention tips
Automated cell counter with slides or hemocytometer
13‐ml clear, round‐bottom, polypropylene, screw cap tubes, 101‐mm length × 16.5‐mm diameter (Sarstedt, cat. no. 60.541)
Cold room, 4°C
13‐ml tubes adapters for SS‐34 rotor (Thermo Fisher Scientific, cat. no. 75003095)
Sorvall RC6 Plus with SS‐34 rotor or equivalent high‐speed refrigerated centrifuge with fixed‐angle rotor (30° to 34°) for 50‐ml tubes
Transfer pipettes
Cleansing tissue
Tweezers
NanoDrop or spectrophotometer
1.5‐ml Protein LoBind tubes (Eppendorf, cat. no. 0030108116)
Dry ice

#### Cell harvest for native ChIP‐seq (CENP‐A)

1a Culture at least 3–4 × 10^7^ cells.
2a Centrifuge the cells 5 min at 360 × *g*, 4°C, in a swinging‐bucket centrifuge. Wash the cells twice with 5 ml ice‐cold 1× PBS using 10‐ml pipettes and pipette aid.
  For adherent cells, aspirate the medium, wash the cells twice with ice‐cold 1× PBS, and detach by gentle scraping.
3a Centrifuge the cells 5 min at 360 × *g*, 4°C, in a swinging‐bucket centrifuge. Remove the supernatant and place the cell pellet on ice.
  From this point onward, perform everything on ice. Proceed to step 9.

#### Cell harvest for cross‐linked ChIP‐seq (CENP‐C) and cross‐linking

1b Culture at least 3–4 × 10^7^ cells.
2b Prepare fresh 10% formaldehyde and 10× glycine (1.375 M) solutions.
3b Add fresh 10% formaldehyde to each culture dish to a final concentration of 1%, swirling to mix.
4b Incubate for 10 min at room temperature.
5b Add fresh 10× glycine to a final 1× concentration, swirling to mix.
6b Incubate for 5 min at room temperature.
7b Centrifuge the cells 5 min at 360 × *g*, 4°C, in a swinging‐bucket centrifuge. Wash the cells twice with 5 ml ice‐cold 1× PBS.
  For adherent cells, aspirate the medium, wash the cells twice with ice‐cold 1× PBS, and detach by gentle scraping.
8b Centrifuge the cells 5 min at 360 × *g*, 4°C, in a swinging‐bucket centrifuge. Remove the supernatant and place the cell pellet on ice.
  From this point onward, perform everything on ice.

#### Cell counting

9 Flick the pellets to disperse the cells and gently combine all cells into a 50‐ml tube. Bring the volume up to 30 ml with ice‐cold 1× PBS.
10 Using a pipette with low‐retention tips, remove 10 µl of cells and add to 10 µl of Trypan blue (1:2 cell dilution).
11 Load 10 µl of the Trypan blue/cell mixture into an automated cell counter slide and estimate cell viability. If >80%, proceed. Otherwise, the cells are not healthy and should be discarded.
12 Based on the total number of viable cells, determine the number of sucrose gradient tubes required.
  The buffer volumes below are sufficient for up to 3–4 × 10^7^ cells.
13 Centrifuge cells 5 min at 360 × *g*, 4°C, in a swinging‐bucket centrifuge.
14 While centrifuging, prepare aliquots of buffers I, II, and III and supplement them with DTT, PMSF, and a protease inhibitor (PI) cocktail. For every 3–4 × 10^7^ cells, prepare: 2 ml buffer I, 2 ml buffer II, and 8 ml buffer III.
  Add the following to buffers I, II, and III fresh:

**Table:** 

Final concentration	Dilution	Stock concentration	Volume for 2 ml	Volume for 8 ml
0.5 mM DTT	1:2000	1 M DTT	1 µl	4 µl
0.1 mM PMSF	1:1000	100 mM PMSF	2 µl	8 µl
0.1× PI cocktail	1:1000	100× PI Cocktail	2 µl	8 µl
15 Pipette 8 ml of supplemented buffer III into the bottom of a 13‐ml Sarstedt tube on ice.
16 After centrifugation, pour off the supernatant (PBS) and flick the pellet to resuspend the cells. Keep the cells on ice.
  From this point onward, perform everything in a 4°C cold room.

#### Nuclei isolation

17 Add 2 ml (per 3–4 × 10^7^ cells) of ice‐cold supplemented buffer I, then gently pipette up and down 4 times.
18 Add 2 ml (per 3–4 × 10^7^ cells) of ice‐cold supplemented buffer II, then invert the tube twice. Incubate cells on ice for no more than 10 min.
  NP‐40 in buffer II permeabilizes the plasma membrane.
  Optional: To assess permeabilization efficiency, mix 10 µl of cells with 10 µl of Trypan blue. Pipette the suspension onto a microscope slide and check under a light microscope to ensure that most nuclei are intact and that there are minimal intact cells.
19 At the end of the 10‐min incubation, slowly layer the cell suspension onto the supplemented buffer III in Sarstedt tubes.
20 Place the Sarstedt tubes in an adapter in an SS‐34 rotor and centrifuge 20 min at 10,000 × *g*, 4°C, with acceleration = 10 and deceleration = 5.
  Depending on the centrifuge it might be needed to simply specify “high” acceleration and “slow” deceleration.
21 Meanwhile, aliquot 1 to 2 ml of buffer IV, supplement it, and keep it on ice.

**Table:** 

Final concentration	Dilution	Stock concentration	Volume for 2 ml
0.5 mM DTT	1:2000	1 M DTT	1 µl
0.1 mM PMSF	1:1000	100 mM PMSF	2 µl
0.1× PI cocktail	1:1000	100× PI Cocktail	2 µl
22 After centrifugation, the nuclei should be pelleted at the bottom of the tube, and the cytoplasmic components should be adhered to the wall of the tube or floating in the upper layer (Fig. 2). If this separation is observed, place the tubes on ice and go back to the cold room.

**Figure 2:**
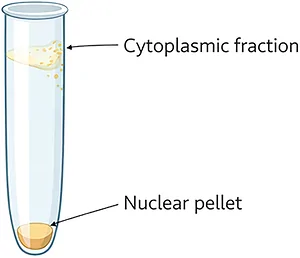
Separation of nuclei from cytoplasmic components upon ultracentrifugation. Representative schematic of the tube after ultracentrifugation, showing cytoplasmic components adhering to the inner wall of the upper layer of the tube and a compact nuclear pellet at the bottom. Proper separation is critical for efficient recovery of intact nuclei and minimization of cytoplasmic contamination in downstream ChIP‐seq analyses.

23 Use a transfer pipette to carefully remove the top sucrose layer and most of the second sucrose layer, moving the pipette in a circular motion around the periphery of the sucrose layers and avoiding bubbles.
24 Take a cleansing tissue, wrap it around the tweezers, and wipe the inner walls of the tubes to remove residual sucrose. Repeat 2 to 3 times.
25 Remove the remaining sucrose layers with a new transfer pipette. Use a new cleansing tissue wrapped around tweezers to remove the remaining sucrose at the bottom of the tube without disturbing the pellet.
26 Carefully add 200 µl of supplemented buffer IV to the bottom of the tube.
27 Resuspend the nuclei in supplemented buffer IV by shaking. Do not pipette or vortex.
28 Measure chromatin concentration by taking an OD_260_ reading using a NanoDrop or spectrophotometer.
  If necessary, dilute an aliquot of nuclei to obtain accurate readings; however, do not dilute more than 0.5 to 1 ml (or not less than 400 to 500 ng/µl)
29 Aliquot 150 µg of chromatin in labeled 1.5‐ml Protein LoBind tubes. Aliquot an additional 20 to 150 µg of chromatin in a labeled 1.5‐ml Protein LoBind tube to use for the MNase titration, as described in Support Protocol 1 and/or for the optimization of sonication conditions, as described in Critical Parameters in the Commentary section.
30 Snap‐freeze aliquots on dry ice and store at –80°C. Proceed to Basic Protocol 2.

## Support Protocol 1 TITRATION OF MNase

This support protocol describes the optimization of micrococcal nuclease (MNase) digestion conditions for native centromeric chromatin preparations. Because the extent of nuclease digestion strongly influences nucleosome recovery and downstream immunoprecipitation efficiency, titration of MNase is an essential preliminary step prior to processing experimental samples. The goal is to identify the enzyme concentration that yields predominantly mononucleosomal DNA fragments, typically 145 to 160 bp, while minimizing both under‐ and over‐digestion. Titration of MNase should be performed for every newly processed sample.

### Materials

20 to 150 µg chromatin aliquot (from Basic Protocol 1)
Buffer IV, ice‐cold (see recipe)
1 M DTT (Thermo Fisher Scientific, cat. no. R0861)
100 mM PMSF (Thermo Fisher Scientific, cat. no. 36978)
100× Halt protease inhibitor (PI) cocktail (Thermo Fisher Scientific, cat. no. 87786)
100 mM CaCl_2_ (Current Protocols, 2006)
MNase (Thermo Fisher Scientific, cat. no. 88216)
0.5 M EGTA (Current Protocols, 2006)
QIAquick PCR purification kit for PCR clean‐up (Qiagen, cat. no. 28104)
High‐sensitivity Bioanalyzer kit (Agilent, cat. no. 5067‐4626) or 1.5% agarose gel

Pipettes and low‐retention tips
1.5‐ml Protein LoBind tubes (Eppendorf, cat. no. 0030108116)
Heating block (Thermo Fisher Scientific, cat. no. 88871001)
Agilent 2100 Bioanalyzer

1 Thaw a frozen aliquot of 20 to 150 µg chromatin on ice.
2 Dilute chromatin to 400 ng/µl with ice‐cold buffer IV (supplemented with DTT, PMSF, and PI cocktail) using a cut pipette tip.
3 Test MNase concentrations of 0.025, 0.05, 0.1, 0.15, and 0.2 U/µg chromatin. Since this results in 5 different reactions, divide the chromatin aliquot equally into five 1.5‐ml Protein LoBind tubes.
4 Add CaCl_2_ to a final concentration of 3 mM.
5 Add MNase to each of the tubes.
6 Flick tube to mix, pulse‐spin (5 s at 500 × *g*, room temperature), and then incubate at 37°C for 10 min on a heating block.
7 Add EGTA to a final concentration of 10 mM and flick to mix.
  This will inhibit the MNase.
8 Incubate the tube on ice for 5 min.
9 PCR purify with QIAquick PCR purification kit for PCR clean‐up according to the manufacturer's protocol and eluting in 30 µl EB buffer (supplied with kit).
10 Dilute each sample to 5 to 10 ng/µl and assess fragment size using a high‐sensitivity Bioanalyzer kit and Bioanalyzer according to the manufacturer's instructions or by running a 1.5% agarose gel.
  The ideal result is a peak at 145 to 160 bp that has (1) no shoulders on either side, and (2) no peaks or only very small peaks at 50 to 100 bp. Determine the optimal amount of MNase based on this.

## Basic Protocol 2 MNase DIGESTION AND IMMUNOPRECIPITATION OF CENP‐A CHROMATIN

This protocol describes the controlled digestion of native centromeric chromatin with micrococcal nuclease (MNase), followed by extraction and immunoprecipitation of CENP‐A‐containing chromatin complexes. Under native conditions, MNase digestion releases predominantly mono‐ and oligonucleosomal fragments while preserving the stable association of CENP‐A with centromeric DNA. Following nuclease digestion, soluble chromatin is extracted from nuclei by low‐ and high‐salt incubation steps, and the soluble fraction enriched in centromeric nucleosomes is subsequently immunoprecipitated using a CENP‐A‐specific antibody. An aliquot of the soluble chromatin is retained as input control for downstream enrichment analysis and normalization.

### Materials

150 µg chromatin aliquot (from Basic Protocol 1)
Buffer IV, ice‐cold (see recipe)
1 M DTT (Thermo Fisher Scientific, cat. no. R0861)
100 mM PMSF (Thermo Fisher Scientific, cat. no. 36978)
100× Halt protease inhibitor (PI) cocktail (Thermo Fisher Scientific, cat. no. 87786)
100 mM CaCl_2_ (Current Protocols, 2006)
MNase (Thermo Fisher Scientific, cat. no. 88216)
0.5 M EGTA (Current Protocols, 2006)
10 mM EDTA, pH 8 (Current Protocols, 2006)
5 M NaCl (Current Protocols, 2006)
TE buffer (Current Protocols, 2006)
1.5% agarose gel
Buffer V with and without Tween (see recipe)
50% slurry of pre‐washed NET‐blocked Protein G Sepharose beads (see Support Protocol 2)
CENP‐A antibody (Enzo, cat. no. ADI‐KAM‐CC006)

Ice bucket and ice
Pipettes and low‐retention tips
Refrigerated swinging‐bucket centrifuge (Sorvall ST4 Plus, Thermo Fisher Scientific, cat. no. 75009525)
Heating block (Thermo Fisher Scientific, cat. no. 88871001)
Rotator mixer (Invitrogen, cat. no. 15920D)
NanoDrop or spectrophotometer
1.5‐ml Protein LoBind tubes (Eppendorf, cat. no. 0030108116)

#### MNase digestion and chromatin extraction from nuclei

1 Take a frozen aliquot of 150 µg of chromatin and thaw on ice.
2 Supplement an aliquot of ice‐cold buffer IV, adding the following:

**Table:** 

Final concentration	Dilution	Stock concentration	Volume for 2 ml
0.5 mM DTT	1:2000	1 M DTT	1 µl
0.1 mM PMSF	1:1000	100 mM PMSF	2 µl
0.1× PI cocktail	1:1000	100× PI Cocktail	2 µl
3 Dilute chromatin to 400 ng/µl with ice‐cold supplemented buffer IV with a cut pipette tip.
4 Add CaCl_2_ to a final concentration of 3 mM.
5 Add MNase to the tube at the appropriate concentration (determined in Support Protocol 1; usually 0.05 to 0.5 U/µg chromatin).
6 Flick the tube to mix, pulse‐spin (5 s at 500 × *g*, room temperature), and then incubate at 37°C for 10 min on a heating block.
7 Add EGTA to a final concentration of 10 mM.
  This will inhibit the MNase.
8 Incubate on ice for 5 min and remove 10 µl as a total extract (TEX) sample.
9 Centrifuge 7 min at 500 × *g*, 4°C, in a swinging‐bucket centrifuge.
10 Remove the supernatant (S1) and take a gel sample (10 µl).
11 Flick the pellet to disperse nuclei. Add the starting volume of 10 mM EDTA, pH 8, to the pellet and flick.
  At least 250 µl of volume are needed to get inversion of cells during the next step.
12 Incubate for 2 hr while rotating at 4°C. Make sure the solution is inverting during rotation or chromatin will not be extracted efficiently from the nuclei. This may be helped by a slower rotation.
13 Add 5 M NaCl to a final concentration of 0.5 M. Do this dropwise and mix by inverting.
14 Incubate for 45 min while rotating at 4°C.
15 Meanwhile, supplement an aliquot of TE buffer, adding the following:

**Table:** 

Final concentration	Dilution	Stock concentration	Volume for 2 ml
0.5 mM DTT	1:2000	1 M DTT	1 µl
0.1 mM PMSF	1:1000	100 mM PMSF	2 µl
0.1× PI cocktail	1:1000	100× PI Cocktail	2 µl
16 Spin 5 min at maximum speed, 4°C.
17 Collect the supernatant (S2) in a labeled 1.5‐ml Protein LoBind tube and take a gel sample (10 µl).
18 Resuspend the pellet (P) in the starting volume of the supplemented TE buffer and take a gel sample (10 µl).
19 Measure the OD_260_ of TEX, S1, S2, and P using a NanoDrop or spectrophotometer. Calculate percent of chromatin in S2:
$$\%=\frac{S2}{\left(S1+S2+P\right)}$$
  This number should ideally be ≥35%.
20 Run a 1.5% agarose DNA gel to ensure that most of the chromatin is in the S2.
  If all of this looks good, proceed to the next step. Otherwise, start over.

#### Immunoprecipitation

21 Supplement an aliquot of buffer V with Tween and an aliquot of buffer V without Tween, adding the following:

**Table:** 

Final concentration	Dilution	Stock concentration	Volume for 2 ml
0.5 mM DTT	1:2000	1 M DTT	1 µl
0.1 mM PMSF	1:1000	100 mM PMSF	2 µl
0.1× PI cocktail	1:1000	100× PI Cocktail	2 µl
22 Dilute S2 to 100 ng/µl with supplemented buffer V with Tween.
23 Cut a low‐retention pipette tip and add 50 µl of a 50% slurry of pre‐washed Protein G Sepharose beads.
24 Rotate for 20 min at 4°C.
25 Centrifuge for 5 min at 3000 × *g*, 4°C, then remove supernatant.
26 Save 50 µl of chromatin as Input. Add 100 µl of supplemented buffer V without Tween and store at −80°C.
  If chromatin volume is limited, a minimum of 10 µl can be reserved as Input DNA. The volume should then be adjusted to 150 µl with buffer V.
27 Add 20 µl (20 µg) of CENP‐A antibody to S2.
28 Incubate overnight at 4°C with rotation in the rotator mixer. Then proceed to Basic Protocol 3.

## Alternate Protocol 1 CHROMATIN SONICATION AND IMMUNOPRECIPITATION OF CENP‐C CHROMATIN COMPLEXES

In this protocol, nuclei isolated in Basic Protocol 1 are subjected to controlled sonication to fragment cross‐linked chromatin into sizes suitable for immunoprecipitation (IP) and downstream sequencing. Following fragmentation, the chromatin is cleared of debris by centrifugation. The resulting chromatin fraction is then immunoprecipitated using antibodies against CENP‐C during overnight rotation to maximize antibody binding. An aliquot of fragmented chromatin is recovered as input material before antibody incubation to serve as a reference for genomic background and normalization against which peaks will be called in the data analysis (i.e., input).

### Additional Materials (also see Basic Protocol 2)

2× sonication buffer (see recipe)
CENP‐C antibody (Abcam, cat. no. ab50974)

Covaris tubes (Covaris, cat. no. 520045)
M220 Focused‐ultrasonicator (Covaris, cat. no. 500295)
1.5‐ml Protein LoBind tubes (Eppendorf, cat. no. 0030108116)
Refrigerated microcentrifuge (MicroCL 21R, Thermo Fisher Scientific, cat. no. 75002561)

#### Sonication

1 Take two frozen chromatin aliquots for each antibody and thaw them on ice.
2 Meanwhile, supplement an aliquot of 2× sonication buffer according to the needed volume (same as chromatin), adding the following:

**Table:** 

Final concentration	Dilution	Stock concentration	Volume for 2 ml
0.5 mM DTT	1:2000	1 M DTT	1 µl
0.1 mM PMSF	1:1000	100 mM PMSF	2 µl
0.1× PI cocktail	1:1000	100× PI Cocktail	2 µl
3 Combine the chromatin into a single tube, add an equal volume of 2× sonication buffer, and place on ice.
4 Aliquot the sample into Covaris tubes, using 100 µl per tube. Place on ice.
5 Sonicate chromatin using a Covaris sonicator with the following parameters:
  5 min
  5% Duty Factor (DF)
  4°C
  Light OFF.
6 Combine the sonicated chromatin back to 1.5‐ml Protein LoBind tubes and centrifuge 10 min at 9600 × *g*, 4°C.
7 Meanwhile, supplement an aliquot of buffer V with Tween, adding the following:

**Table:** 

Final concentration	Dilution	Stock concentration	Volume for 2 ml
0.5 mM DTT	1:2000	1 M DTT	1 µl
0.1 mM PMSF	1:1000	100 mM PMSF	2 µl
0.1× PI cocktail	1:1000	100× PI Cocktail	2 µl
8 Transfer the supernatant to a new labeled 1.5‐ml Protein LoBind tube. This is the cross‐linked chromatin preparation.

#### Immunoprecipitation

9 Dilute to 100 ng/µl with buffer V supplemented with Tween and divide into at least 2 tubes per antibody.
  To allow proper inversion during incubation, the optimal volume per tube is 0.5 to 1 ml.
10 Save 10 µl of chromatin as Input and store at –20°C.
11 For each CENP‐C IP tube, add 100 µl (10 µg) of CENP‐C antibody.
12 Incubate overnight at 4°C with rotation in the rotator mixer. Then proceed to Alternate Protocol 2.

## Support Protocol 2 NET BLOCKING AND WASHING OF PROTEIN G SEPHAROSE BEADS

This protocol describes the preparation of NET‐blocked, pre‐washed Protein G Sepharose beads for use in native chromatin immunoprecipitation. Blocking and washing steps reduce nonspecific binding of chromatin and proteins to the beads, thereby improving signal‐to‐noise ratio during immunoprecipitation. The procedure yields a 50% bead slurry equilibrated in supplemented buffer V with Tween, suitable for immediate use. Excess prepared beads can be stored at 4°C for short‐term use.

### Materials

Protein G Sepharose beads (Cytiva, cat. no. 17‐0618‐01)
NET buffer (see recipe)
TE buffer (Current Protocols, 2006)
20% sodium azide (Current Protocols, 2006)
Buffer V with Tween (see recipe)
1 M DTT (Thermo Fisher Scientific, cat. no. R0861)
100 mM PMSF (Thermo Fisher Scientific, cat. no. 36978)
100× Halt protease inhibitor (PI) cocktail (Thermo Fisher Scientific, cat. no. 87786)

Rotating mixer (Invitrogen, cat. no. 15920D)
Refrigerated centrifuge (MicroCL 21R, Thermo Fisher Scientific, cat. no. 75002561)
1.5‐ and 15‐ml tubes (Eppendorf, cat. no. 0030123158; Corning, cat. no. 352097)
Pipette and low‐retention filtered tips

#### NET blocking of Protein G Sepharose beads

1 Add 1.5 ml of Protein G Sepharose beads to 10 ml NET buffer.
2 Rotate for 1 hr, at 4°C.
3 Wash the beads three times with TE buffer as follows:
  a. Add 10 ml of TE buffer.
  b. Rotate for 5 min at 4°C.
  c. Centrifuge for 5 min at 340 × *g*, 4°C.
4 Resuspend in 4.5 ml of TE buffer, and add sodium azide to final concentration of 0.03%, totaling 6 ml.
5 Add 1.5‐ml aliquots to tubes and store at 4°C.

#### Washing of NET‐blocked beads

6 Supplement an aliquot of TE and an aliquot of buffer V with Tween, adding the following:

**Table:** 

Final concentration	Dilution	Stock concentration	Volume for 2 ml
0.5 mM DTT	1:2000	1 M DTT	1 µl
0.1 mM PMSF	1:1000	100 mM PMSF	2 µl
0.1× PI cocktail	1:1000	100× PI Cocktail	2 µl
7 Add 1 ml of supplemented TE buffer to the NET‐blocked beads.
8 Rotate for 5 min at 4°C.
9 Centrifuge 5 min at 340 × *g*, 4°C, and remove supernatant.
10 Repeat steps 7 to 9.
11 Add 1 ml of supplemented buffer V with Tween to beads.
12 Rotate for 5 min at 4°C.
13 Centrifuge 5 min at 340 × *g*, 4°C, and remove supernatant.
14 Resuspend the beads to 50% slurry with 250 µl supplemented buffer V with Tween and keep on ice.

## Basic Protocol 3 DNA EXTRACTION

This protocol describes the recovery and purification of DNA from the immunoprecipitated CENP‐A‐containing chromatin complexes obtained in Basic Protocol 2. Protein G Sepharose beads were selected for the native workflow because they provide efficient recovery of stable CENP‐A‐containing nucleosomal complexes under gentle rotation and wash conditions. Following overnight antibody incubation and bead capture, the chromatin complexes are extensively washed to reduce nonspecific background, and the associated DNA is released by proteinase K digestion. DNA is then purified by phenol/chloroform/isoamyl alcohol extraction followed by column‐based clean‐up. The corresponding input sample is processed in parallel to provide a matched control for downstream library preparation, enrichment assessment, and sequencing analysis.

### Materials

50% slurry of pre‐washed NET‐blocked Protein G sepharose beads on ice (see Support Protocol 2)
Buffer V with and without Tween (see recipe)
1 M DTT (Thermo Fisher Scientific, cat. no. R0861)
100 mM PMSF (Thermo Fisher Scientific, cat. no. 36978)
100× Halt protease inhibitor (PI) cocktail (Thermo Fisher Scientific, cat. no. 87786)
TE buffer (Millipore, cat. no. 8890)
10% high purity sodium dodecyl sulfate (SDS) (Current Protocols, 2006)
Proteinase K (Qiagen, cat. no. 19131)
Proteinase K stop mix (see recipe)
RNase A (Qiagen, cat. no. 19101)
Phenol/chloroform/isoamyl alcohol (25:24:1 v/v/v) (Invitrogen, cat. no. 15593031)
QIAquick PCR purification kit for PCR clean‐up (Qiagen, cat. no. 28104)
MinElute PCR purification kit (Qiagen, cat. no. 28004)
High‐sensitivity Qubit kit (Thermo Fisher Scientific, cat. no. Q32854)

Pipettes and low‐retention filtered tips
Rotator mixer (Invitrogen, cat. no. 15920D)
Refrigerated microcentrifuge (MicroCL 21R, Thermo Fisher Scientific, cat. no. 75002561)
1.5‐ml Protein LoBind tubes (Eppendorf, cat. no. 0030108116)
Ice bucket and ice
Heating block (Thermo Fisher Scientific, cat. no. 88871001)
Vortex
2‐ml DNA LoBind tubes (Eppendorf, cat. no. 022431048)
Qubit tubes (Thermo Fisher Scientific, cat. no. Q32856)
Qubit (Invitrogen, cat. no. Q33238)

#### Antibody capture with protein G beads

1 Cut a low‐retention tip and add 100 µl of a 50% slurry of pre‐washed Protein G Sepharose beads.
2 Rotate for 3 hr at 4°C.
3 Centrifuge 5 min at 2400 × *g*, 4°C.
4 Meanwhile, supplement an aliquot of buffer V with Tween, and an aliquot of buffer V without Tween, adding the following:

**Table:** 

Final concentration	Dilution	Stock concentration	Volume for 2 ml	Volume for 4 ml
0.5 mM DTT	1:2000	1 M DTT	1 µl	2 µl
0.1 mM PMSF	1:1000	100 mM PMSF	2 µl	4 µl
0.1× PI cocktail	1:1000	100× PI Cocktail	2 µl	4 µl
5 Remove supernatant carefully without disturbing the beads.
6 Wash the beads with three times with 1 ml of buffer V supplemented with Tween:
  a. Slowly pipette the buffer from the bottom upward to resuspend the sample.
  b. Incubate on ice for 5 min.
  c. Centrifuge for 5 min at 2400 × *g*, 4°C.
  d. Remove the supernatant.
7 Cut a low‐retention tip and add 500 µl of supplemented buffer V without Tween to the beads by gently pipetting from the bottom upward to ensure complete resuspension of the beads.
8 Transfer the beads to a new labeled 1.5‐ml Protein LoBind tube.
9 Using a fresh tip, add 500 µl of supplemented buffer V without Tween to the original tube. Use the previously used tip to rinse the walls of the tube and transfer the wash to the new tube containing the beads, ensuring complete recovery.
10 Incubate beads on ice for 5 min.
11 Centrifuge 5 min at 2400 × *g*, 4°C.
12 Transfer the supernatant carefully without disturbing the beads in a new labeled 1.5‐ml Protein LoBind tube. Discard the remaining beads.

#### DNA extraction

13 For the ChIP sample:
  a. Add 300 µl of TE buffer.
  b. Add high purity SDS to a final concentration of 0.5%.
  c. Add proteinase K (500 µg/ml final concentration).
  d. Incubate for 4 hr at 56°C on a heating block.
14 For the input:
  a. Add the same volume of proteinase K stop mix (150 µl).
  b. Add 1 µl of RNase A diluted at 10 mg/ml.
  c. Incubate for 1 hr at 37°C.
  d. Add 12 µl of proteinase K diluted at 2.5 mg/ml (100 µg/ml final concentration).
  e. Incubate for 3 hr at 37°C.
15 After the incubation, add 300 µl of phenol:chloroform:isoamyl alcohol to the tubes.
16 Vortex, and spin for 10 min at 13,800 × *g*, room temperature.
  The recovered volume is 200 to 300 µl.
17 Transfer the aqueous phase to a new labeled 2‐ml DNA LoBind tube.
18 PCR purify all the samples.
  Use the MinElute PCR purification kit for the ChIP sample according to the manufacturer's protocol and elute in 10 µl of buffer EB. Use the QIAquick PCR purification kit for the Input sample, according to the manufacturer's protocol and eluting in 30 µl buffer EB.
19 Quantify the samples and Input using the Qubit and Qubit high‐sensitivity assay according to the manufacturer's protocol. Store the samples at −20°C. Proceed to Basic Protocol 4.

## Alternate Protocol 2 REVERSAL OF CROSS‐LINKS AND DNA PURIFICATION

This protocol describes the recovery and purification of DNA from immunoprecipitated chromatin complexes obtained under cross‐linked conditions, following the overnight antibody incubation described in Alternate Protocol 1. Chromatin complexes are first captured using protein G beads and subjected to a series of wash steps to minimize nonspecific background. The use of a commercial immunoprecipitation kit notably accelerates the capture of immunoprecipitated chromatin while introducing very low levels of non‐specific binding. In contrast to the native workflow, which uses Protein G Sepharose beads for recovery of stable nucleosomal complexes, the cross‐linked workflow employs Dynabeads Protein G magnetic beads to facilitate rapid and reproducible capture of fragmented chromatin during multiple wash and decrosslinking steps. Magnetic separation also minimizes sample loss and improves handling consistency for low‐input cross‐linked samples. Cross‐links are then reversed to release DNA associated with centromere‐bound proteins such as CENP‐C, followed by purification with AMPure XP beads. The selected bead‐to‐sample ratio preferentially retains DNA fragments larger than ∼200 bp, thereby reducing the contribution of short fragments that can decrease library complexity and increase background signal in downstream ChIP‐seq analyses. The corresponding input sample is processed in parallel to provide a matched control for normalization and downstream data analysis.

### Additional Materials (also see Basic Protocol 3)

Dynabeads Protein G immunoprecipitation kit (Invitrogen, cat. no. 10007D)
2× decrosslinking buffer (see recipe)

Dynamag magnet (Life Technologies, cat. no. 12321D)
Benchtop centrifuge (MicroCL 21, Thermo Fisher Scientific, cat.no. 75002549)
Thermomixer (Eppendorf, cat. no. 2231001208)

Additional reagents and equipment for AMPure XP bead clean‐up (see Support Protocol 3)

#### Antibody capture with protein G beads

1 For each immunoprecipitation tube, transfer 50 µl of Dynabeads Protein G to a new labeled 1.5‐ml Protein LoBind tube and place it on the magnet.
2 Remove the supernatant from the beads, then remove the tubes from the magnet.
3 Add the chromatin‐antibody mixture from the overnight immunoprecipitation to the beads and mix well by pipetting.
4 Rotate at room temperature for 30 min in the rotator mixer.
5 Pulse‐spin (5 s at 500 × *g*, room temperature) to collect the volume to the bottom of the tube.
6 Wash beads three times with 200 µl Wash buffer (contained in the kit):
  a. Place tubes on the magnet and let them stand for 5 min.
  b. Remove the supernatant.
  c. Remove the tubes from the magnet, add 200 µl of Wash buffer, and mix well by pipetting.
7 Resuspend the beads in 100 µl of Wash buffer, then transfer the entire volume to a new labeled 1.5‐ml Protein LoBind tube.
8 Place tubes on the magnet and remove the supernatant.

#### Reversal of cross‐linking

9 For each ChIP sample, add to each tube:
  a. 20 µl Elution buffer (from the kit)
  b. 20 µl of 2× decrosslinking buffer
  c. 5 µl proteinase K
  d. Mix well by pipetting to resuspend beads.
10 Retrieve the Input from the −20°C storage and add:
  a. 10 µl Elution buffer (from the kit)
  b. 20 µl of 2× decrosslinking buffer
  c. 5 µl proteinase K
  d. Mix well by pipetting.
11 Incubate at 65°C, 800 rpm in the thermomixer for 1 hr.
12 Place bead‐bound samples on the magnet and transfer the supernatant to a new set of labeled 1.5‐ml Protein LoBind tubes.
13 Purify all samples and Input with 1.6× volume of AMPure XP (72 µl beads), as described in Support Protocol 3. Elute in 12 µl of 10 mM Tris·HCl in a new set of 1.5‐ml DNA LoBind tubes.
  At this step, pool all tubes corresponding to the same antibody for each sample. The final combined volume must be 12 µl. For example, if you have four tubes, elute each tube with 3 µl and then combine the four eluates into a single tube to obtain a final volume of 12 µl.
14 Quantify the samples and Input using the Qubit High‐sensitivity assay according to the manufacturer's protocol, with up to 2 µl of sample. Store the samples at –20°C. Proceed to Basic Protocol 4.

## Support Protocol 3 BEAD CLEAN‐UP

This protocol describes the purification of DNA using AMPure XP beads. Bead‐based clean‐up is used at multiple stages to remove enzymes, salts, and reaction components while retaining DNA fragments of the appropriate size for ChIP‐seq libraries. Careful handling, thorough bead resuspension, and complete ethanol removal are essential to maximize recovery and reproducibility.

### Materials

AMPure XP beads (Beckman, cat. no. A63881)
Absolute ethanol, molecular biology grade (Fisher Scientific, cat. no. BP28184)
UltraPure DNase/RNase‐free distilled water (Invitrogen, cat. no. 10977015)
10 mM Tris·HCl, pH 7.5 (Current Protocols, 2006)

Pipettes and low‐retention filtered tips
Mini microcentrifuge (Corning, cat. no. 6770)
Dynamag magnet (Life Technologies, cat. no. 12321D)
1.5‐ml DNA LoBind tubes (Eppendorf, cat. no. 0030108051)

#### Clean‐up

1 Let the beads, ethanol, and water aliquots re‐equilibrate at room temperature for at least 30 min.
2 Resuspend the re‐equilibrated beads by inverting and tapping the tube. Ensure the beads are fully resuspended before adding to the sample. Do not vortex or spin the beads.
3 Add the required volume of AMPure XP beads to each tube.
4 Incubate for 10 min. In the meantime, prepare a fresh 70% ethanol dilution.
5 Pulse‐spin the tubes and place the tubes on the magnet. Wait for 5 min.
6 Remove the supernatant.
  To avoid bead loss, leave some of the volume behind.
7 With the tubes still on the magnet, add 100 µl of freshly prepared 70% ethanol and allow it to stand for 30 s.
8 Remove the supernatant, then repeat the 70% ethanol wash once for a total of two washes.
9 Remove the ethanol and air‐dry the beads on the magnet for 10 min.
  It is critical to remove as much of the ethanol as possible. To do so, remove the ethanol in the first pipetting step, spin down to collect the excess ethanol at the bottom, place the tubes back on the magnet, and remove the remaining ethanol in the second pipetting step. Be sure all the ethanol has evaporated before proceeding.
10 Resuspend the beads in the required volume of 10 mM Tris·HCl.
11 Incubate for 5 min.
12 Place the tubes in the magnet and let them stand for 2 min.
13 Transfer all the eluate volumes to a new set of labeled 1.5‐ml DNA LoBind tubes.

## Basic Protocol 4 LIBRARY PREPARATION AND SIZE SELECTION

This protocol describes the construction of Illumina sequencing platform‐compatible libraries from ChIP and input DNA using a low‐input library preparation kit, with minor adjustments to accommodate the limited DNA quantities typically obtained from centromeric ChIP‐seq experiments. The workflow includes end repair, adapter ligation, PCR amplification, and clean‐up steps optimized for ultra‐low input DNA. The use of library preparation enhancers and high‐fidelity enzymes supports efficient adapter ligation and amplification while minimizing amplification bias and adapter dimer formation. Following amplification, libraries undergo gel‐based size selection to enrich for fragments of ∼330 bp (including adapters), corresponding to ∼210 bp inserts. This size range is chosen to balance efficient cluster generation with optimal mapping performance in repetitive genomic regions. Removal of shorter fragments and adapter dimers reduces background signal and improves the reliability of read alignment and quantitative assessment of centromeric enrichment.

### Materials

ChIP samples (from Support Protocol 3)
10 mM Tris·HCl, pH 7.5 (Current Protocols, 2006)
Ovation Ultralow kit (Tecan, cat. no. 0344NB‐32)
32‐plex adapter plate (Tecan, cat. no. S02317‐FG)
High‐sensitivity Qubit kit (Thermo Fisher Scientific, cat. no. Q32854)
50 bp DNA ladder (Invitrogen, cat. no. 10488099)
2% E‐Gel agarose gel with SYBR Gold Safe Stain (Invitrogen, cat. no. G402022)
5% bleach
Zymoclean Gel DNA recovery kit (Zymo, cat. no. D4008)
Absolute ethanol, molecular biology grade (Fisher Scientific, cat. no. BP28184)
High‐sensitivity Bioanalyzer kit (Agilent, cat. no. 5067‐4626)

Pipette and low‐retention filtered tips
0.2‐ml PCR strip tubes (Thermo Fisher Scientific, cat. no. AB2000)
Vortex (Corning, cat. no. 6774)
Mini microcentrifuge (Corning, cat. no. 6770)
Ice bucket and ice
Thermal cycler (Bio‐Rad, cat. no. 1861096)
1.5‐ml DNA LoBind tubes (Eppendorf, cat. no. 0030108051)
Qubit tubes (Thermo Fisher Scientific, cat. no. Q32856)
Qubit (Invitrogen, cat. no. Q33238)
E‐Gel Power Snap electrophoresis device (Invitrogen, cat. no. G8100)
Gel imager
Spatula
Sterile scalpel blades
High‐precision balance
Tweezers
Heating block (Thermo Fisher Scientific, cat. no. 88871001)
Benchtop centrifuge (MicroCL 21, Thermo Fisher Scientific, cat. no. 75002549)
Agilent 2100 Bioanalyzer
Additional reagents and equipment for AMPure XP bead clean‐up (see Support Protocol 3)

#### End‐repair

1 For library preparation, transfer 10 µl of each ChIP sample into 0.2 ml PCR tubes. Dilute 1 µl of Input DNA with 9 µl of 10 mM Tris·HCl in a 0.2‐ml PCR tube.
  If possible, adjust the initial volume of all samples so that library preparation starts with the same concentration for all samples. Bring the final volume up to 10 µl with 10 mM Tris·HCl as needed.
2 Retrieve the Ovation Ultralow end repair buffer mix (blue: ER1), end repair enzyme mix (blue: ER2), end repair enhancer (blue: ER3), and nuclease‐free water (green: D1) from –20°C storage.
3 Thaw ER1 and D1 at room temperature. Mix by vortexing, pulse‐spin, and place on ice.
4 Pulse‐spin the contents of ER2 and ER3 and place them on ice.
5 Prepare a master mix by combining ER1, ER2, and ER3 in a 0.2‐ml tube at the volumes listed below. Mix by pipetting and place on ice.

**Table:** 

Component	For 1 sample	For 3.5 samples
End repair buffer mix (blue: ER1)	3.5 µl	12.25 µl
End repair enzyme mix (blue: ER2)	0.5 µl	1.75 µl
End repair enhancer (blue: ER3)	1.0 µl	3.5 µl
6 Add 5 µl of the end repair master mix to each sample tube.
7 Mix by pipetting, pulse‐spin, and place on ice.
8 Place the tubes in a pre‐warmed thermal cycler programmed to run the following program:
  25°C for 30 min
  70°C for 10 min
  Hold at 4°C.
9 Remove the tubes from the thermal cycler, spin to collect condensation, and place on ice.
10 Continue immediately with the adapter ligation step.

#### Adapter ligation

11 Retrieve the Ovation Ultralow ligation buffer mix (yellow: L1), ligation adapter mix (yellow: L2), and ligation enzyme mix (yellow: L3) from –20°C storage.
12 Thaw L1 and L2 on ice. Mix by vortexing, pulse‐spin, and place on ice.
13 Pulse‐spin down L3 and place on ice.
14 Add to each sample 3 µl of Adapters (Adapter plate L2V26 Unique Dual Index).
15 Make a master mix by combining D1, L1, and L3 in a 0.2‐ml tube according to the volumes shown below. Mix by pipetting and place on ice.
  The L1 ligation buffer mix is very viscous; be sure to pipette slowly to avoid introducing bubbles. Use the master mix immediately.

**Table:** 

Component	For 1 sample	For 3.5 samples
Water (green: D1)	1.5 µl	5.25 µl
Ligation buffer mix (yellow: L1)	6 µl	21 µl
Ligation enzyme mix (yellow: L3)	1.5 µl	5.25 µl
16 Add 9 µl of the ligation master mix to each tube. Mix thoroughly by pipetting slowly and gently, pulse‐spin, and place on ice. Proceed immediately with the incubation.
17 Place the tubes in a pre‐warmed thermal cycler programmed to run the following program:
  25°C for 30 min
  70°C for 10 min
  Hold at 4°C.
18 Remove the tubes from the thermal cycler, pulse‐spin to collect condensation, and transfer all the samples to a new set of labeled 1.5‐ml DNA LoBind tubes.
19 Purify the ligation with 0.8× volumes of AMPure XP beads (80 µl) as described in Support Protocol 3. Elute in 35 µl of 10 mM Tris·HCl and transfer all the eluate to a new 0.2‐ml tube.

#### Library amplification

20 Retrieve the Ovation Ultralow amplification buffer mix (red: P1), amplification primer mix (red: P2), and amplification enzyme mix (red: P3) from –20°C storage.
21 Thaw P1, P2, and P3 at room temperature. Mix by vortexing, pulse‐spin, and place on ice.
22 Spin P3 and place on ice.
23 Make a master mix by sequentially combining P1 and P2 in a 0.2‐ml tube, using the volumes shown below. Add P3 at the last moment, mix well by pipetting, taking care to avoid bubbles, then pulse‐spin and place on ice.

**Table:** 

Component	For 1 sample	For 3.5 samples
Amp buffer mix (red: P1 ver 7)	12.75 µl	44.62 µl
Amp primer mix (red: P2 ver 5)	1.25 µl	4.37 µl
Amp enzyme mix (red: P3 ver 4)	1 µl	3.5 µl
24 On ice, add 15 µl of the amplification master mix to each sample.
25 Place the tubes in a pre‐warmed thermal cycler programmed to run the following program:
  72°C for 2 min
  95°C for 3 min
  15 cycles of 98°C for 20 s, 65°C for 30 s, and 72°C for 30 s
  72°C for 1 min
  Hold at 4°C.
26 Remove the tubes from the thermal cycler, pulse‐spin to collect condensation, and transfer all the samples to a new set of labeled 1.5‐ml DNA LoBind tubes.
27 Purify the amplified library with 1 volume of AMPure XP beads (50 µl) as described in Support Protocol 3. Elute in 33 µl of 10 mM Tris·HCl.
28 Quantify the Library using the Qubit High‐sensitivity assay with 1 µl of library per sample, then proceed to size selection. Store the library at –20°C.

#### Size selection

29 For each ChIP sample and Input DNA, combine at least 10 µl of the library with 10 mM Tris·HCl up to 20 µl in a new 1.5‐ml DNA LoBind tube.
30 Dilute 2 µl of 50 bp DNA ladder with 18 µl of 10 mM Tris·HCl.
31 Load 20 µl of the diluted ladder and all the samples into a 2% E‐Gel EX agarose gel with SYBR Gold Safe Stain. Start the E‐Gel EX program at 1% to 2% for 10 min.
  To ensure a correct run, load all the wells without samples with 20 µl of 10 mM Tris·HCl.
32 Confirm proper sample migration and acquire an image of the gel.
33 Open the gel with a spatula or a knife and place it on the gel imager.
34 Cut the ∼300 bp band with a scalpel blade and transfer it to a new 1.5‐ml DNA LoBind tube using tweezers.
  Clean the scalpel blade and tweezers with 5% bleach between samples.
35 Weigh the collected gel slice using a balance.
36 Briefly spin down the tubes to collect all gel slices at the bottom.
37 For each tube, add the volume of buffer ADB (from the Zymoclean kit) corresponding to 3× the weight and incubate the mixture at 55°C in a heating block for ∼10 min. Mix several times during incubation by vortexing and spinning.
38 Pulse‐spin the tube and transfer the melted gel slices to a prelabeled Zymo‐spin column inserted into a collection tube.
39 In a benchtop centrifuge, spin 30 s at maximum speed, room temperature.
40 Wash the column twice with 200 µl of fresh 80% ethanol, and centrifuge 1 min at 21,000 × *g* (i.e., maximum speed), room temperature.
41 Transfer the column to a clean 1.5‐ml DNA LoBind tube and let it dry for 1 min.
42 Add 7.5 µl of pre‐warmed 10 mM Tris·HCl directly to the column matrix. Let it stand for 1 min and then centrifuge 1 min at 21,000 × *g* (i.e., maximum speed), room temperature, to collect the eluate.
43 Repeat step 42.
  Adjust the elution volume based on the library concentration. Avoid diluting the library >2 ng/µl. A two‐step dilution is preferred to maximize the yield.
44 Assess the quality and quantity of the size‐selected libraries using the Qubit high‐sensitivity assay and Bioanalyzer according to the manufacturer's instructions. Use 1 µl of the library each.
45 If applicable, proceed to satellite DNA enrichment evaluation, as described in Support Protocol 4. Store at –20°C until sequencing, then proceed to Basic Protocol 5.

## Support Protocol 4 qPCR TO ASSESS α‐SATELLITE DNA ENRICHMENT

This protocol describes a quantitative PCR (qPCR)‐based assessment of centromeric α‐satellite DNA enrichment in ChIP samples relative to input DNA. The α‐satellite primer pair, originally described by Alonso et al. (2007), amplifies a conserved region of the α‐satellite monomer encompassing the CENP‐B box, a sequence motif widely conserved among primates and many other mammals (Kipling & Warburton, 1997). Because this target sequence is present in numerous α‐satellite arrays, the assay provides a genome‐wide readout of bulk centromeric chromatin enrichment rather than a locus‐specific measurement. Given the highly repetitive, multi‐copy nature of these sequences, this assay serves as a pre‐sequencing quality‐control checkpoint rather than a quantitative measurement of enrichment at individual centromeres. The Aldolase A gene was chosen as a control because it is a single‐copy euchromatic locus unrelated to centromeric chromatin, providing a reference for non‐specific background signal.

### Materials

ChIP DNA libraries (from Basic Protocol 4)
Primers (see Table 1)
SupRealQ Ultra Hunter SYBR qPCR Master Mix (U+) (Vazyme, cat. no. 10007D)
UltraPure DNase/RNase‐free distilled water (Invitrogen, cat. no. 10977015)

Pipette and low‐retention tips
Optical 96‐well reaction plates (Applied Biosystems, cat. no. N8010560)
Optical sealing film for PCR plates (Applied Biosystems, cat. no. 4311971)
Mini plate spinner centrifuge (Axygen, cat. no. PLATESPINNER‐EU)
Ice bucket and ice
Plate vortex (Fisher Scientific, cat. no. 02‐217‐024)
qPCR machine (Applied Biosystems, cat. no. A28574)

**Table 1:** qPCR Primer Sequences and Targets

Target	Forward primer	Reverse primer
α‐satellite	TTTCGTTGGAAACGGGA	CGTGCTCTGTGAAAGGGAAT
Aldolase‐A	TCCTGGCAAGATAAGGAGTTGAC	ACACACGATAGCCCTAGCAGTTC

#### Standard curve generation

1 For each primer pair, a standard curve should be generated by doing serial dilutions of template DNA. From the standard curve, the primers' efficiency can be calculated.
  Most qPCR software can automatically determine primer pair efficiency when running standard curve samples. Suggested dilutions of template DNA to use are: 0.002, 0.02, 0.2, 0.1, 1, 2, 10, and 20 ng.

#### qPCR

2 Set up the plate with 3 replicates and 3 negative controls (NTC) for each primer/DNA combination.
3 Prepare a 2 ng/µl dilution of all the template DNA.
4 Prepare a 10 mM primer mix for each pair.
5 Pipette 1 µl of the 2 ng/µl dilution of the template DNA in each assigned well and 1 µl of water in the NTC wells. Temporarily seal the plate, pulse‐spin, and place on ice.
6 For each primer pair, prepare a reaction mix as follows:

**Table:** 

Component	For 1 sample	For 3 samples ± NTC
Master mix	5 µl	60 µl
10 mM primer mix	2 µl	24 µl
H_2_O	2 µl	24 µl
7 Add 9 µl of reaction mix to all the wells. Carefully seal the plate, vortex, pulse‐spin, and place on ice.
8 Place the plate in the qPCR machine and run the following program:
  94°C for 2 min (initial denaturation)
  40 cycles of 94°C for 15 s (denaturation), 58°C for 20 s (annealing), and 72°C for 1 min (extension)
  72°C for 5 min (final extension)
  4°C (hold)

#### Fold enrichment analysis

9 Determine the fold enrichment of the ChIP samples relative to the Input as follows:
$$\text{Fold-enrichment}=X^{\Delta \text{CT}}$$
  Where, CT = cycle at which fluorescence is above the threshold (threshold set by qPCR software), ∆CT = CTmean Input – CTmean ChIP, and X = 1 + primer efficiency.

## Basic Protocol 5 ChIP‐seq DATA PROCESSING AND ANALYSIS

This protocol outlines a representative workflow for processing and analyzing centromeric ChIP‐seq data, including read trimming, alignment, normalization, and visualization. Data analysis of centromeric regions requires careful consideration of mapping parameters, increasing the minimum seed length to account for lower read variability and the maximum seed occurrences to account for a higher number of similar reads, as in the case of repetitive DNA. Moreover, normalization strategies and strict filters on read mapping quality are crucial for removing mismapped reads, which are due to the repetitive nature of centromeric DNA. The described approach emphasizes relative enrichment and reproducibility.

### Materials

Sickle v1.33 (Joshi & Fass, 2011)
TrimGalore v0.6.10 (Krueger et al., 2023)
Bwa mem v0.7.18 (Li, 2013)
Samtools v1.21 (Danecek et al., 2021)
bamCompare v3.5.6 (Ramírez et al., 2014)
Genome browsers/viewers, e.g., UCSC (https://genome‐euro.ucsc.edu/index.html) or IGV (https://igv.org/)

#### Data quality control and filtering

1 Trim low‐quality 5’ or 3’ of reads with sickle, while removing all reads smaller than 100 bp:


sickle pe ‐t sanger ‐q 20 ‐l 100 ‐f <input_R1.fastq> ‐r <input_R2.fastq> ‐o <QualityTrimmed_R1.fastq> ‐p <QualityTrimmed_R2.fastq> ‐s <QualityTrimmed_singlets.fastq>

2 Trim adapters with TrimGalore:


trim_galore –paired –retain_unpaired –length 100 –illumina –stringency 5 –fastqc <QualityTrimmed_R1.fastq> <QualityTrimmed_R2.fastq>

#### Alignment

3 Align to the indexed assembly or reference genome with bwa mem:


bwa mem ‐t 8 ‐k 50 ‐c 1000000 <assembly.fa> <QualityTrimmed_AdapterTrimmed_R1.fastq> <QualityTrimmed__AdapterTrimmed_R2.fastq>

4 Remove unmapped reads and not‐primary alignments; sort and index bam files with samtools:


samtools view ‐bS ‐F 2308 | samtools sort ‐m 8G ‐T tmp1 ‐o <output.bam>samtools index <output.bam>

#### Normalization and peak calling

5 Call peaks by normalizing and comparing read numbers in bins with bamCompare, for reads with at least 1 of mapping quality:


bamCompare ‐b1 <ChIP.bam> ‐b2 <Input.bam> –operation ratio –binSize 5000 –minMappingQuality 1 ‐o <ChIPvsInput.bw>

6 Visualize the bigWig file in genome browsers or genome viewers, such as UCSC or IGV.

## REAGENTS AND SOLUTIONS

*See Current Protocols (2006) for recipes of commonly used reagents that can be used for the recipes below, unless otherwise indicated*.

### Buffer I

For 50 ml (make up remaining volume with H_2_O):
5.47 g sucrose (0.32 M)
1 ml of 3 M KCl (60 mM)
0.75 ml of 1 M Tris·HCl, pH 7.5 (15 mM)
0.15 ml of 5 M NaCl (15 mM)
0.25 ml of 1 M MgCl_2_ (5 mM)
0.01 ml of 0.5 M EGTA (0.1 mM)
Filter‐sterilize using a 0.22‐µm filter
Store up to 6 months at 4°C
Immediately before using add:
0.5 mM DTT (1:2000 dilution from 1 M stock)
0.1 mM PMSF (1:10000 dilution from 1 M stock)
0.1× protease inhibitor (PI) cocktail (1:1000 dilution from 100× stock; Thermo Fisher Scientific, cat. no. 87786)

### Buffer II

For 50 ml (make up remaining volume with H_2_O):
5.47 g sucrose (0.32 M)
1 ml of 3 M KCl (60 mM)
0.75 ml of 1 M Tris·HCl, pH 7.5 (15 mM)
0.15 ml of 5 M NaCl (15 mM)
0.25 ml of 1 M MgCl_2_ (5 mM)
0.01 ml of 0.5 M EGTA (0.1 mM)
0.125 to 0.5 ml of 20% NP‐40 (0.05% to 0.2%; Thermo Fisher Scientific, cat. no. 85124)
Filter‐sterilize using a 0.22‐µm filter
Store up to 6 months at 4°C
Immediately before using add:
0.5 mM DTT (1:2000 dilution from 1 M stock)
0.1 mM PMSF (1:10000 dilution from 1 M stock)
0.1× protease inhibitor (PI) cocktail (1:1000 dilution from 100× stock; Thermo Fisher Scientific, cat. no. 87786)

### Buffer III

For 50 ml (make up remaining volume with H_2_O):
20.53 g sucrose (1.2 M)
1 ml of 3 M KCl (60 mM)
0.75 ml of 1 M Tris·HCl, pH 7.5 (15 mM)
0.15 ml of 5 M NaCl (15 mM)
0.25 ml of 1 M MgCl_2_ (5 mM)
0.01 ml of 0.5 M EGTA (0.1 mM)
Filter‐sterilize using a 0.22‐µm filter
Store up to 6 months at 4°C
Immediately before using add:
0.5 mM DTT (1:2000 dilution from 1 M stock)
0.1 mM PMSF (1:10000 dilution from 1 M stock)
0.1× protease inhibitor (PI) cocktail (1:1000 dilution from 100× stock; Thermo Fisher Scientific, cat. no. 87786)

### Buffer IV

For 50 ml (make up remaining volume with H_2_O):
5.81 g sucrose (0.34 M)
1 ml of 3 M KCl (60 mM)
0.75 ml of 1 M HEPES, pH 7.4 (15 mM)
0.15 ml of 5 M NaCl (15 mM)
0.2 ml of 1 M MgCl_2_ (4 mM)
Filter‐sterilize using a 0.22‐µm filter
Store up to 6 months at 4°C
Immediately before using add:
0.5 mM DTT (1:2000 dilution from 1 M stock)
0.1 mM PMSF (1:10000 dilution from 1 M stock)
0.1× protease inhibitor (PI) cocktail (1:1000 dilution from 100× stock; Thermo Fisher Scientific, cat. no. 87786)

### Buffer V with or without Tween

For 50 ml (make up remaining volume with H_2_O):
1 ml of 1 M Tris·HCl, pH 8 (20 mM)
0.5 ml of 0.5 M EDTA (5 mM)
5 ml of 5 M NaCl (500 mM)
0.5 ml of 20% Tween‐20 (0.2%) (omit for buffer V without Tween)
Filter‐sterilize using a 0.22‐µm filter
Store up to 6 months at 4°C
Immediately before using add:
0.5 mM DTT (1:2000 dilution from 1 M stock)
0.1 mM PMSF (1:10000 dilution from 1 M stock)
0.1× protease inhibitor (PI) cocktail (1:1000 dilution from 100× stock; Thermo Fisher Scientific, cat. no. 87786)

### Decrosslinking buffer, 2×

For 10 ml (make up remaining volume with H_2_O):
1 ml of 1 M Tris·HCl, pH 8.0 (100 mM)
2 ml of 0.5 M EDTA (100 mM)
1.2 ml of 5 M NaCl (600 mM)
0.5 ml of 20% SDS (1%)
Filter‐sterilize using a 0.22‐µm filter
Store up to 6 months at room temperature

### NET buffer

For 10 ml (make up remaining volume with H_2_O):
0.3 ml of 5 M NaCl (150 mM)
0.5 ml of 1 M Tris·HCl, pH 7.5 (50 mM)
0.02 ml of 0.5 M EDTA (1 mM)
50 µl of 20% NP‐40 (0.1%; Thermo Fisher Scientific, cat. no. 85124)
2.5 ml of 1% gelatin (1 g in 10 ml of boiling dH_2_O; Sigma‐Aldrich, cat. no. G1890)
15 µl of 20% sodium azide
Filter‐sterilize using a 0.22‐µm filter
Store up to 6 months at 4°C

### Proteinase K stop mix

For 5 ml (make up remaining volume with H_2_O):
0.6 ml of 5 M NaCl (0.6 M)
0.2 ml of 0.5 M EDTA (20 mM)
0.1 ml of 1 M Tris·HCl, pH 7.5 (20 mM)
1 ml of 5% SDS (1%)
Prepare fresh

### Sonication buffer, 2×

For 50 ml (make up remaining volume with H_2_O):
4.5 ml of 1 M HEPES, pH 7.9 (90 mM)
5 ml of 5 M NaCl (500 mM)
1 ml of 0.5 M EDTA (10 mM)
1 ml Triton X‐100 (1%)
0.5 ml of 20% Tween‐20 (0.2%)
Prepare fresh
Immediately before using add:
0.5 mM DTT (1:2000 dilution from 1 M stock)
0.1 mM PMSF (1:10000 dilution from 1 M stock)
0.1× protease inhibitor (PI) cocktail (1:1000 dilution from 100× stock; Thermo Fisher Scientific, cat. no. 87786)

## COMMENTARY

### Critical Parameters

The choice between native and cross‐linked ChIP‐seq is a critical experimental parameter and should be determined by the centromeric target of interest. Cross‐linking of chromatin is essential for preserving protein–DNA interactions involving non‐histone centromeric components, such as CENP‐C and other kinetochore‐associated proteins. In contrast, CENP‐A can be efficiently analyzed under native conditions because it is stably incorporated into centromeric nucleosomes and remains tightly associated with DNA.

Efficient enrichment of centromeric chromatin depends on maintaining chromatin integrity during nuclei isolation and achieving consistent chromatin fragmentation prior to immunoprecipitation. The sequential lysis and sucrose gradient centrifugation steps described in Basic Protocol 1 are critical for removing cytoplasmic contaminants while preserving intact nuclei. Nuclear disruption or incomplete separation of sucrose layers can reduce immunoprecipitation efficiency and increase background signal. Strict temperature maintenance at 4°C throughout these steps is therefore essential.

Chromatin fragmentation must be tightly controlled and depends on the selected workflow. For native ChIP‐seq, MNase digestion conditions must be carefully optimized to generate predominantly mononucleosomal fragments while preserving centromeric nucleosome integrity. MNase titration should therefore be performed for each new sample type or cell line, as described in Support Protocol 1. The ideal digestion profile consists of a major peak 145 to 160 bp, with minimal discrete bands corresponding to di‐ and three‐nucleosomes, as shown in Figure 3. On the other hand, for cross‐linked samples, sonication parameters have been optimized through systematic condition testing. For each condition, 10 µl of chromatin was diluted 1:1 with 2× sonication buffer and sonicated varying sonication time and power (duty factor 5%, Fig. 4A; duty factor 15%, Fig. 4B). Following post‐sonication centrifugation (Alternate Protocol 1), the supernatant was reverse‐cross‐linked (Alternate Protocol 2) and analyzed on a 1.5% agarose gel. The selected condition was subsequently validated using a 100 µl chromatin aliquot to confirm that fragmentation remained optimal at larger chromatin amounts.

**Figure 3:**
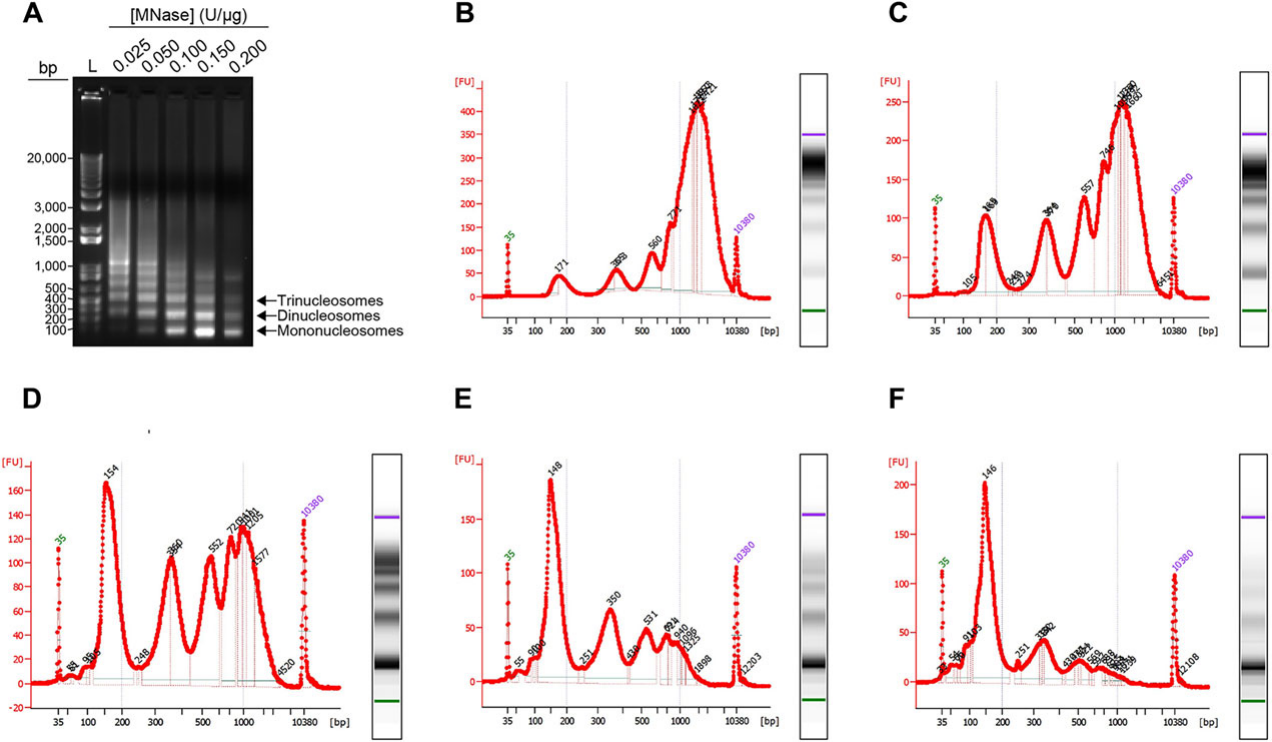
Optimization of MNase digestion conditions for native CENP‐A ChIP‐seq. Representative agarose gel (**A**) and corresponding Bioanalyzer traces obtained after digestion with increasing MNase concentrations (0.025, 0.05, 0.10, 0.15, and 0.20 U/µg) (**B**‐**F**) are shown. The optimal digestion condition (0.15 U/µg) produced a predominant mononucleosomal peak (145 to 160 bp) while minimizing di‐ and trinucleosomal fragments.

**Figure 4:**
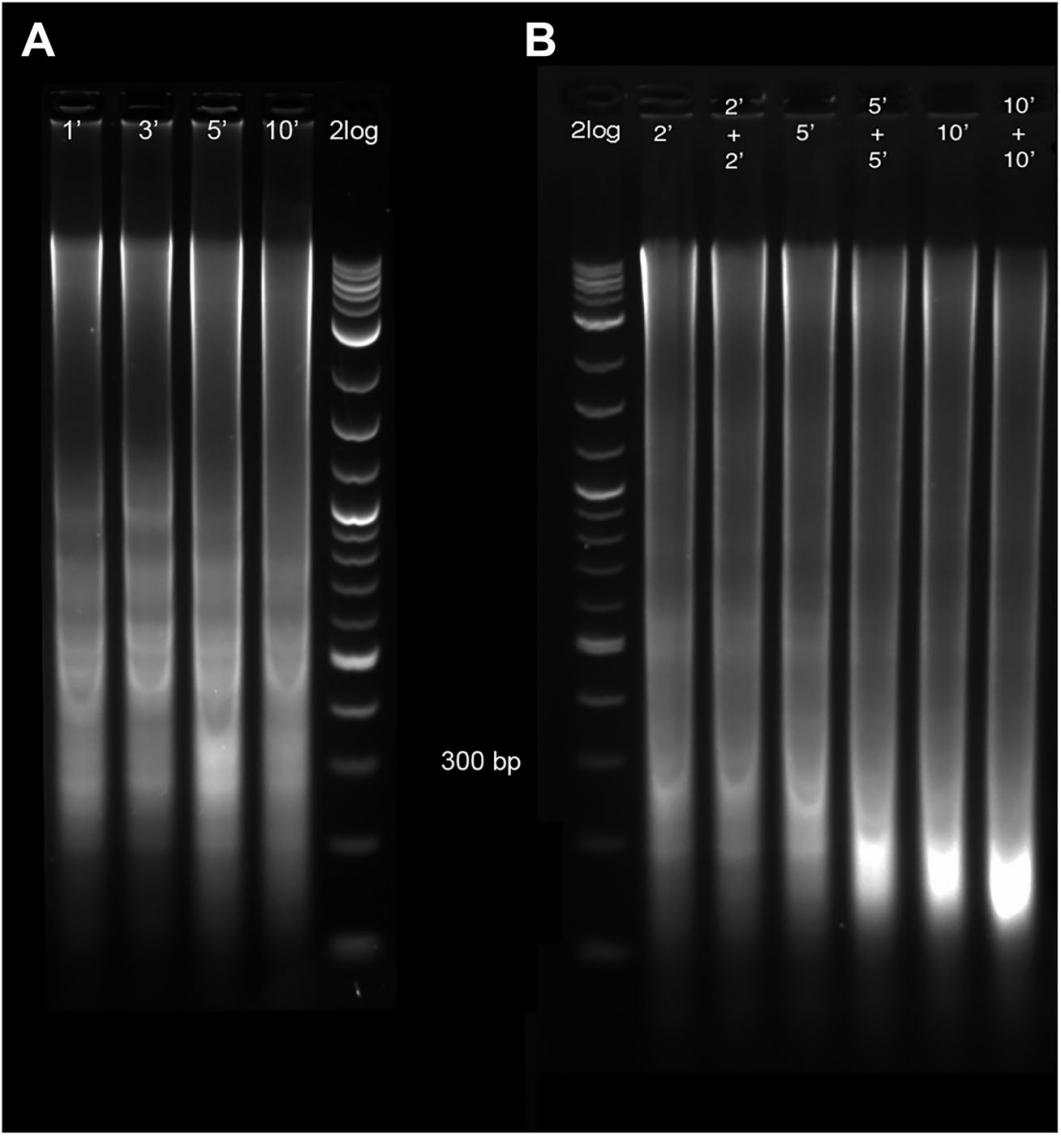
Optimization of chromatin sonication conditions. Representative 1.5% agarose gel showing chromatin fragment distributions obtained after sonication under the tested Covaris conditions. For each condition, 10 µl of chromatin was diluted 1:1 with 2× sonication buffer, sonicated, and the recovered supernatant was reverse‐cross‐linked prior to gel analysis. (**A**) Sonication performed at a 5% duty factor for 1, 3, 5, and 10 min. (**B**) Sonication profiles obtained at a 15% duty factor after 2 min, two 2‐min pulses separated by a 1‐min incubation on ice, 5 min, two 5‐min pulses separated by a 1‐min incubation on ice, 10 min, and two 10‐min pulses separated by a 1‐min incubation on ice. The 2‐log DNA ladder is shown, with the 300 bp band indicated.

Antibody performance is a major determinant of success for centromeric targets. Antibodies against centromere proteins, such as CENP‐A or CENP‐C, should be validated for ChIP and independently verified by immunofluorescence prior to sequencing. The antibodies used in this protocol (CENP‐A, Enzo, cat. no. ADI‐KAM‐CC006; CENP‐C, Abcam, cat. no. ab50974) have been successfully applied to ChIP experiments in human and non‐human primate cell lines. Inclusion of Tween‐20 in the buffers is essential and must not be omitted, as it reduces nonspecific binding to beads during immunoprecipitation and enhances proteinase K activity. For cross‐linked samples, careful preparation of the decrosslinking buffer is also required. Because it contains 100 mM Tris·HCl, its concentration must be reduced by half as specified here to avoid inhibition of proteinase K, which would otherwise compromise protein digestion during cross‐link reversal.

Finally, downstream analysis parameters strongly influence data interpretation. Genome assemblies used for alignment should include sequence representations or annotations that permit mapping of centromere‐derived reads. Mapping and filtering settings should minimize loss of informative multimapping reads while limiting nonspecific signals. In this protocol, this balance is achieved by setting the “minMappingQuality” parameter to 1 during peak calling with bamCompare, thereby excluding non‐primary alignments while retaining informative reads.

### Troubleshooting

Centromeric ChIP‐seq is sensitive to multiple steps in the workflow, and minor deviations can lead to substantial reductions in enrichment or increased background signal. Many common issues arise from incomplete nuclei isolation, suboptimal chromatin fragmentation, or antibody‐related variability. Because centromeric chromatin constitutes a minor and highly repetitive fraction of the genome, technical issues that might be tolerated in conventional ChIP‐seq experiments can have amplified effects in this context. Table 2 summarizes the most common issues encountered during this protocol and provides practical solutions to address them.

**Table 2:** Troubleshooting the Most Common Technical Issues in the Centromeric ChIP‐seq Protocol

Problem	Likely cause	Specific solution
Low number of cells	Limited starting material; cell lines with poor cell growth or low cell viability	This protocol can be performed with as few as 2 × 10⁷ cells, which is sufficient for a single ChIP experiment; when working at this lower input, minimize sample loss during handling and consider reducing the number of parallel immunoprecipitations
Incompatibility between rotor and tube adapters	Lack of suitable adapters for the rotor; mismatch between tube dimensions and rotor specifications	If compatible adapters are not available, use 30‐ml Nalgene tubes (cat. no. 3119‐0030) as an alternative; in this case, double the number of input cells and all buffer volumes to maintain proper gradient formation and ensure efficient nuclei recovery
Low recovery of nuclei	Incomplete separation during the sucrose gradient	Verify layer formation after centrifugation; avoid disturbing interfaces when removing sucrose
Poor chromatin fragmentation	MNase digestion or sonication conditions not optimized	Re‐optimize MNase titration (native workflow) or confirm Covaris settings and sample volume (crossed‐linked workflow); assess fragment size before IP
Low ChIP DNA yield or low enrichment in centromeric DNA	Inefficient antibody binding	Increase input chromatin; verify antibody specificity by immunofluorescence on metaphase preparations
High background	Nonspecific binding to beads	Add IgG control; consider increasing wash stringency; consider switching to Sepharose beads

### Understanding Results

The results described in this protocol, including the representative ChIP‐seq datasets, were obtained using a lymphoblastoid cell line derived from a *Pongo pygmaeus* individual (PPY17). The sequencing data were aligned to the genome assembly generated from the same cell line (NCBI BioProject ID: PRJNA1433240), as presented in de Gennaro et al. (2026). Successful application of this protocol results in reproducible enrichment of centromeric chromatin relative to input DNA. Achieving this outcome requires careful evaluation of quality metrics at multiple stages.

Nuclei isolation (Basic Protocol 1) can be monitored indirectly by measuring OD_260_. Using 2 × 10⁷ cells as starting material, this protocol typically yields at least four 150‐µg aliquots of chromatin.

Chromatin fragmentation is a critical determinant of success and must be interpreted according to the selected workflow. For the native workflow, successful MNase digestion (Support Protocol 1 and Basic Protocol 2) should yield a predominant DNA peak at 145 to 160 bp, corresponding to mononucleosomal fragments, with minimal shoulders or low‐molecular‐weight peaks (Fig. 3). Because MNase activity is highly dependent on enzyme concentration and incubation conditions, an MNase titration should be performed before processing experimental samples. Representative agarose gels and Bioanalyzer traces obtained from the titration are shown in Figure 3 and illustrate the progressive generation of mononucleosomal fragments with increasing digestion. Over‐digestion must be avoided, as excessive nuclease treatment may destabilize CENP‐A nucleosomes and reduce recovery. Following MNase digestion, centrifugation separates the soluble chromatin fraction (S2) from the insoluble pellet (P), which consists primarily of cellular debris, insoluble nuclear material, and incompletely extracted chromatin. Both the input DNA control and the immunoprecipitated samples are generated exclusively from the soluble S2 fraction. Consequently, enrichment measurements are determined by comparing immunoprecipitated chromatin with the corresponding input derived from the same soluble material, minimizing potential bias arising from chromatin retained in the pellet. Under the extraction conditions described here, only a small proportion of chromatin remains associated with the pellet, while the vast majority is recovered in the S2 fraction, as demonstrated by agarose gel analysis of the TEX, S1, S2, and P fractions (Fig. 5). These results indicate that chromatin extraction is efficient and that retention of centromeric chromatin in the insoluble fraction is unlikely to affect the specificity of downstream ChIP‐seq enrichment.

**Figure 5:**
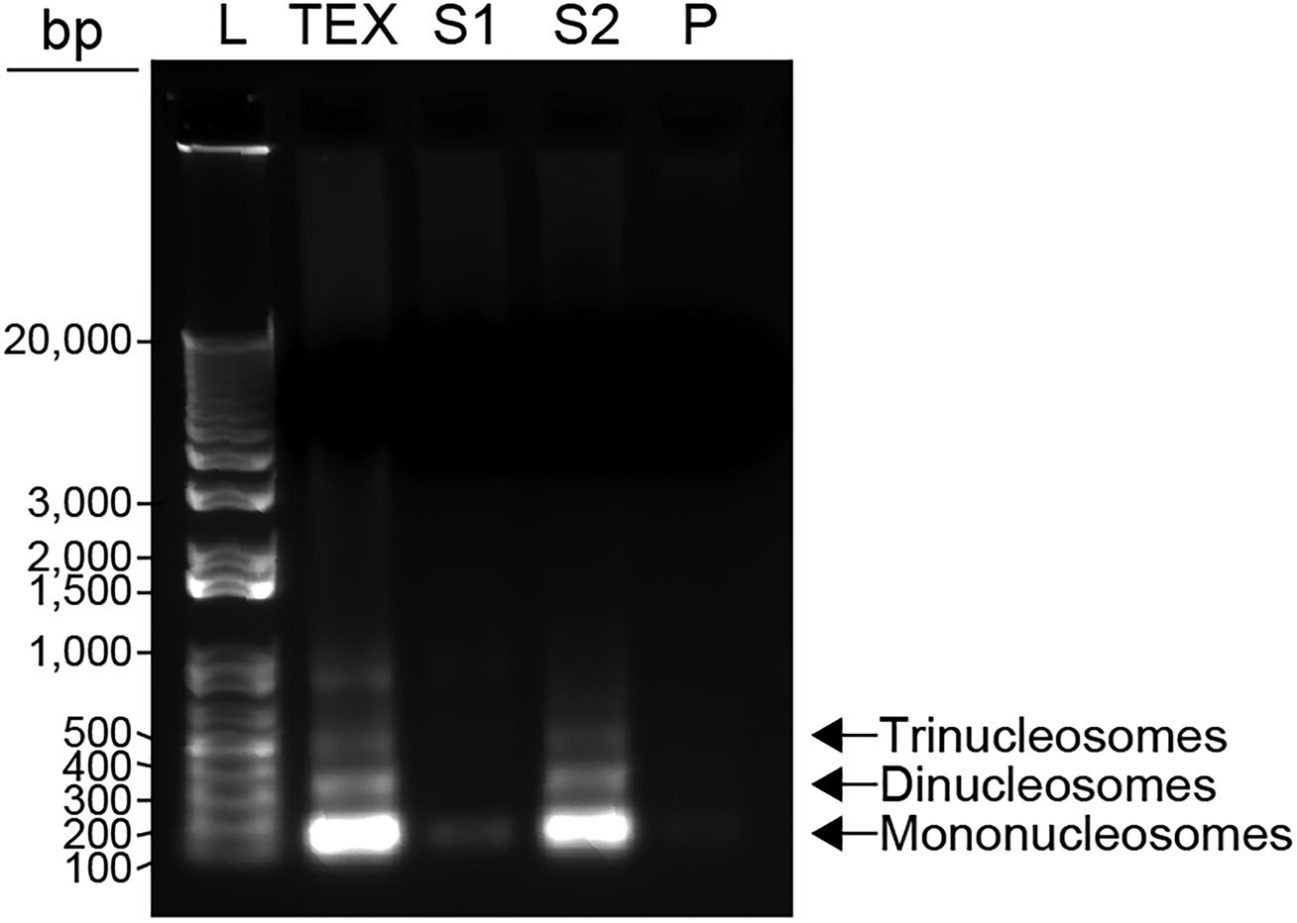
Fractionation of MNase‐digested chromatin during native CENP‐A ChIP. Representative 1.5% agarose gel showing the total extract (TEX), first supernatant (S1), second supernatant (S2), and final pellet (P) fractions following sequential chromatin extraction. The majority of mononucleosomal chromatin is recovered in the S2 fraction, with minimal chromatin remaining in the final pellet (P).

For the cross‐linked workflow, chromatin fragmentation by sonication (Alternate Protocol 1) must be carefully optimized. Over‐shearing should be avoided, as excessive fragmentation can disrupt protein‐DNA complexes involving CENP‐C. The sonication parameters described here have been validated across multiple lymphoblastoid cell lines with consistent results; however, optimization is recommended for new sample types, as discussed in Critical Parameters. Notably, some sonication conditions that produced apparently optimal fragment distributions (e.g., strong enrichment near 200 bp with minimal discrete bands) did not yield detectable α‐satellite enrichment in ChIP samples relative to input by qPCR (Fig. 4B). In contrast, the highest enrichment was obtained under conditions that produced a fragment distribution centered at ∼280 bp, with several discrete bands and a moderate smear (Fig. 4A). Because chromatin domains can differ in their susceptibility to mechanical shearing, the sonication conditions described here were empirically optimized to reproducibly recover CENP‐C‐associated centromeric chromatin while preserving protein–DNA interactions. Although equivalent fragmentation across all genomic regions cannot be assumed, robust enrichment of α‐satellite sequences demonstrates that these conditions efficiently solubilize centromeric chromatin for downstream ChIP‐seq analysis. For applications requiring more complete chromatin fragmentation, the native MNase‐based workflow described in this article may be preferable.

Following antibody incubation and recovery (Basic Protocol 3 for native samples or Alternate Protocol 2 for cross‐linked samples), typical yields are 5 to 10 ng DNA, corresponding to 0.5 to 1 ng/µl by Qubit quantification. Library preparation (Basic Protocol 4) can still be performed when post‐IP DNA concentrations fall below the detection limit of the high‐sensitivity Qubit assay; such samples may produce lower‐concentration libraries but can nevertheless yield high‐quality sequencing data. Libraries generated from 5 to 10 ng input DNA typically produce final concentrations of 40 to 55 ng/µl. Gel‐based size selection at ∼300 bp generally recovers 8% to 10% of input library DNA, although values between 6% and 15% may be observed depending on fragment distribution and handling. Final library concentrations after size selection typically range from 1.8 to 3.0 ng/µl, with an average fragment size of ∼330 bp (Fig. 6).

**Figure 6:**
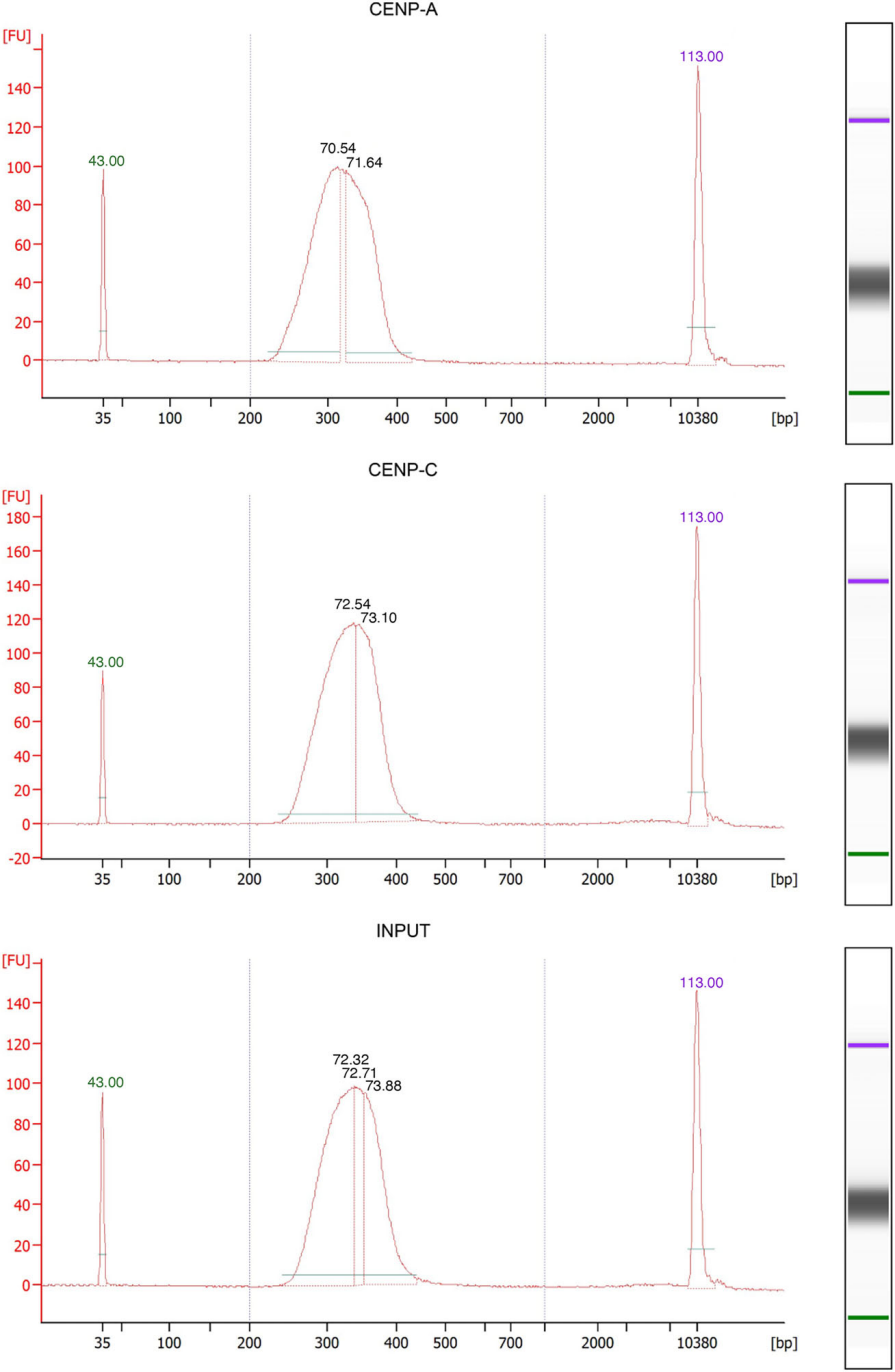
Quality assessment of size‐selected ChIP‐seq libraries by Bioanalyzer analysis. Representative Bioanalyzer electropherograms of final size‐selected libraries generated from CENP‐A ChIP, CENP‐C ChIP, and input DNA following Basic Protocol 4. All libraries display a predominant fragment distribution centered at ∼330 bp, corresponding to ∼210 bp DNA inserts plus sequencing adapters, consistent with the expected size range after gel‐based size selection. Marker peaks are visible at 35 bp and 10,380 bp.

Prior to sequencing, enrichment of α‐satellite DNA should be assessed by qPCR as described in Support Protocol 4. Fold enrichment should be calculated for both the α‐satellite primer pair and the single‐copy control locus (the Aldolase A gene). Successful immunoprecipitation is indicated by enrichment of the α‐satellite target relative to the input, whereas the Aldolase A locus should show little or no enrichment. Although the α‐satellite primers used in this protocol do not amplify all centromeric arrays, they provide a rapid quality‐control checkpoint by confirming preferential recovery of satellite DNA over a non‐centromeric single‐copy locus before sequencing. Primers used here were designed from human sequences but have also been successfully applied to great ape samples. In both cases, robust sequencing results have been obtained with ≥10‐fold enrichment. Acceptable results have also been achieved with ≥4‐fold enrichment in human samples and ≥2‐fold enrichment in great ape samples.

Sequencing libraries generated using this protocol typically produce between 22 and 51 million paired‐end reads after quality filtering, with 96% to 99% of reads successfully aligned to the corresponding reference genome and >99% of aligned reads properly paired. When aligned to the reference assembly generated from the same individual (NCBI BioProject ID: PRJNA1433240), ∼23% of reads pass the mapping quality filter (MAPQ ≥1) used for downstream analysis, as shown in Table 3. This proportion is expected for centromeric ChIP‐seq because α‐satellite arrays are highly repetitive, resulting in a substantial fraction of ambiguously mapped reads that receive MAPQ = 0 and are excluded during analysis. After processing and analysis (Basic Protocol 5), active centromeric regions typically display a peak of ∼200 bp that overlaps between CENP‐A and CENP‐C datasets when reads are aligned to the same genome used for sample generation (Fig. 7). When reads are aligned to a different genome assembly, broader peaks are generally observed.

**Table 3:** Sequencing Summary Statistics and Quality‐Control

Sample	Reads before QC (pairs)	Reads after QC (pairs)	Properly mapped read (pairs)	Reads with MAPQ ≥1
CENP‐A	32,967,590	30,002,457	29,360,158 (99.48%)	13,366,798 (22.6%)
CENP‐C	24,677,213	22,529,102	22,174,839 (99.47%)	10,328,079 (23.2%)
Input	57,083,574	51,236,531	48,957,642 (99.71%)	22,901,335 (23.3%)

**Figure 7:**
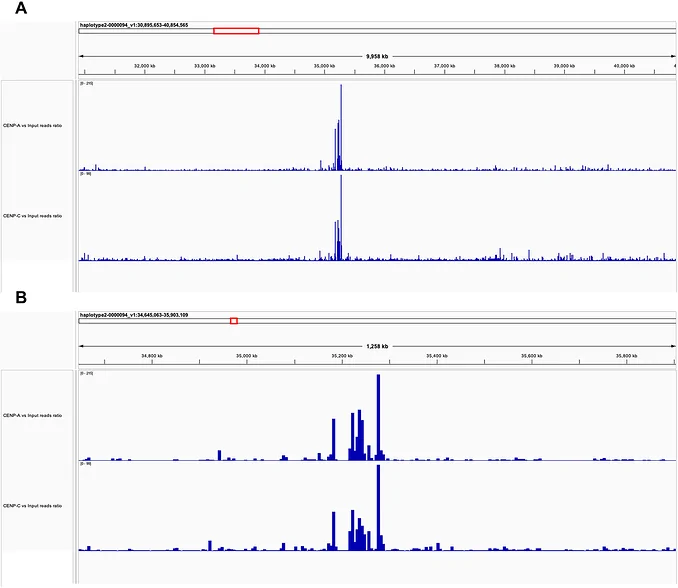
Overlapping enrichment of CENP‐A and CENP‐C at an active centromeric regions. Genome browser view showing ChIP‐seq enrichment profiles for CENP‐A (top) and CENP‐C (bottom) plotted as ChIP/Input ratio tracks across a representative centromeric region. (**A**) Shows the broader genomic context, whereas (**B**) provides a zoomed‐in view of the same region, highlighting the overlapping enrichment peaks. The coincident enrichment of CENP‐A and CENP‐C marks active centromeric chromatin. The red box in (**A**) indicates the genomic region enlarged in the panels below.

### Time Considerations

Each step of this protocol, from Basic Protocol 1 through Basic Protocol 4, can be independently completed within 1 day. An additional day may be required to optimize chromatin fragmentation conditions (see Critical parameters). Support Protocol 4 requires an additional 1 to 2 days if a standard curve for the primers has not yet been generated. Overall, the workflow can typically be completed through library preparation within ∼7 working days.

Under optimized conditions, the wet‐lab portion of the protocol can be condensed to as little as 3 days. In this accelerated schedule, sucrose gradient–based isolation of nuclei (Basic Protocol 1) can be performed on the same day as chromatin digestion or fragmentation and immunoprecipitation (Basic Protocol 2 and Alternate Protocol 1), particularly if sonication or MNase conditions have already been established. Following overnight incubation, DNA extraction (Basic Protocol 3) or reversal of cross‐links and DNA purification (Alternate Protocol 2) can be performed the next day, together with initial library preparation and size selection steps (Basic Protocol 4). Optional stopping points at Basic Protocol 4 include stages immediately before or after PCR amplification and after post‐PCR clean‐up. Pauses should be avoided when instructions specify immediate continuation. The remaining steps of Basic Protocol 4 can then be completed on the same day as qPCR assessment of α‐satellite DNA enrichment (Support Protocol 4).

Data processing and visualization can typically be completed within 2 to 3 days, depending on computational resources.

### Author Contributions

**Alessia Daponte**: Conceptualization; data curation; formal analysis; methodology; writing—original draft; writing—review and editing. **Rachel Schwope**: Conceptualization; methodology. **Caterina** **Manzari**: Methodology; resources. **Lucia Trisolini**: Methodology; resources. **Francesca Antonacci**: Writing—review and editing. **Michele Morgante**: Supervision; writing—review and editing. **Mario Ventura**: Data curation; funding acquisition; writing—review and editing. **Glennis Logsdon**: Conceptualization; methodology; supervision; writing—original draft. **Claudia Catacchio**: Conceptualization; data curation; funding acquisition; supervision; writing—original draft.

### Conflict of Interest

The authors declare no conflict of interest.

## Data Availability

Data sharing is not applicable to this article because it does not report newly generated datasets. The data here presented were obtained from ChIP‐seq experiments generated using the protocol described here and are part of the accompanying manuscript by de Gennaro et al. (2026). The genome assembly used for read alignment is publicly available through NCBI BioProject PRJNA1433240.

## References

[cpz170471-bib-0001] Alonso, A. , Fritz, B. , Hasson, D. , Abrusan, G. , Cheung, F. , Yoda, K. , Radlwimmer, B. , Ladurner, A. G. , & Warburton, P. E. (2007). Co‐localization of CENP‐C and CENP‐H to discontinuous domains of CENP‐A chromatin at human neocentromeres. Genome Biology, 8(7), R148. 10.1186/gb-2007-8-7-r148 PMC2323242 17651496

[cpz170471-bib-0002] Altemose, N. , Logsdon, G. A. , Bzikadze, A. V. , Sidhwani, P. , Langley, S. A. , Caldas, G. V. , Hoyt, S. J. , Uralsky, L. , Ryabov, F. D. , Shew, C. J. , Sauria, M. E. G. , Borchers, M. , Gershman, A. , Mikheenko, A. , Shepelev, V. A. , Dvorkina, T. , Kunyavskaya, O. , Vollger, M. R. , Rhie, A. , … Miga, K. H. (2022). Complete genomic and epigenetic maps of human centromeres. Science, 376(6588), eabl4178. 10.1126/science.abl4178 PMC9233505 35357911

[cpz170471-bib-0003] Current Protocols . (2006). Commonly Used Reagents. Current Protocols in Microbiology, 00, A.2A.1–A.2A.15. 10.1002/9780471729259.mca02as00

[cpz170471-bib-0004] Danecek, P. , Bonfield, J. K. , Liddle, J. , Marshall, J. , Ohan, V. , Pollard, M. O. , Whitwham, A. , Keane, T. , McCarthy, S. A. , Davies, R. M. , & Li, H. (2021). Twelve years of SAMtools and BCFtools. GigaScience, 10(2), giab008. 10.1093/gigascience/giab008 PMC7931819 33590861

[cpz170471-bib-0005] de Gennaro, L. , Yoo, D. , Pistacchia, L. , Magrone, R. , Daponte, A. , Perrone, F. , Ravasini, F. , Mastrorosa, F. K. , Oshima, K. K. , Polano, C. , Hoekzema, K. , Munson, K. M. , Wertz, J. , Marroni, F. , Catacchio, C. R. , Antonacci, F. , Noordermeer, D. , Montinaro, F. , Logsdon, G. A. , … Ventura, M. (2026). A segmental duplication‐mediated deletion leads to neocentromere formation in orangutans. bioRxiv, 2026.04.09.717302. 10.64898/2026.04.09.717302

[cpz170471-bib-0006] Gao, S. , Oshima, K. K. , Chuang, S.‐C. , Loftus, M. , Potapova, T. A. , Montanari, A. , Gordon, D. S. , Yang, Z. , Ashraf, H. , Audano, P. A. , Ayllon, M. , Azov, A. , Balachandran, P. , Basile, A. O. , Beck, C. R. , Bonder, M. J. , Brooks, L. , Byrska‐Bishop, M. , Chaisson, M. J. P. , … Logsdon, G. A. (2026). A global view of human centromere variation and evolution. Nature, 1476–4687. 10.1038/s41586-026-10841-9 PMC13508068 42527608

[cpz170471-bib-0007] Hasson, D. , Panchenko, T. , Salimian, K. J. , Salman, M. U. , Sekulic, N. , Alonso, A. , Warburton, P. E. , & Black, B. E. (2013). The octamer is the major form of CENP‐A nucleosomes at human centromeres. Nature Structural & Molecular Biology, 20(6), 687–695. 10.1038/nsmb.2562 PMC3760417 23644596

[cpz170471-bib-0008] Joshi, N. A. , & Fass, J. N. (2011). *Sickle: A sliding‐window, adaptive, quality‐based trimming tool for FastQ files* (Version Version 1.33) [Computer software]. https://github.com/najoshi/sickle

[cpz170471-bib-0009] Kipling, D. , & Warburton, P. E. (1997). Centromeres, CENP‐B and Tigger too. Trends in Genetics, 13(4), 141–145. 10.1016/S0168-9525(97)01098‐ 9097724

[cpz170471-bib-0010] Krueger, F. , James, F. , Ewels, P. , Afyounian, E. , Weinstein, M. , Schuster‐Boeckler, B. , Hulselmans, G. , & Andrews, S. (2023). *Trim Galore (version 0.6.10)* [Computer software]. 10.5281/zenodo.7598955

[cpz170471-bib-0011] Kumara Mastrorosa, F. , Oshima, K. K. , Rozanski, A. N. , Harvey, W. T. , Eichler, E. E. , & Logsdon, G. A. (2024). Identification and annotation of centromeric hypomethylated regions with Centromere Dip Region (CDR)‐Finder. bioRxiv, 2024.11.01.621587. 10.1101/2024.11.01.621587 PMC11663805 39657946

[cpz170471-bib-0012] Li, H. (2013). *Aligning sequence reads, clone sequences and assembly contigs with BWA‐MEM* (arXiv:1303.3997). arXiv. 10.48550/arXiv.1303.3997

[cpz170471-bib-0013] Logsdon, G. A. , & Eichler, E. E. (2022). The Dynamic Structure and Rapid Evolution of Human Centromeric Satellite DNA. Genes, 14(1), 92. 10.3390/genes14010092 PMC9859433 36672831

[cpz170471-bib-0014] Logsdon, G. A. , Rozanski, A. N. , Ryabov, F. , Potapova, T. , Shepelev, V. A. , Catacchio, C. R. , Porubsky, D. , Mao, Y. , Yoo, D. , Rautiainen, M. , Koren, S. , Nurk, S. , Lucas, J. K. , Hoekzema, K. , Munson, K. M. , Gerton, J. L. , Phillippy, A. M. , Ventura, M. , Alexandrov, I. A. , & Eichler, E. E. (2024). The variation and evolution of complete human centromeres. Nature, 629(8010), 136–145. 10.1038/s41586-024-07278-3 PMC11062924 38570684

[cpz170471-bib-0015] Logsdon, G. A. , Vollger, M. R. , Hsieh, P. , Mao, Y. , Liskovykh, M. A. , Koren, S. , Nurk, S. , Mercuri, L. , Dishuck, P. C. , Rhie, A. , de Lima, L. G. , Dvorkina, T. , Porubsky, D. , Harvey, W. T. , Mikheenko, A. , Bzikadze, A. V. , Kremitzki, M. , Graves‐Lindsay, T. A. , Jain, C. , … Eichler, E. E. (2021). The structure, function and evolution of a complete human chromosome 8. Nature, 593(7857), 101–107. 10.1038/s41586-021-03420-7 PMC8099727 33828295

[cpz170471-bib-0016] McKinley, K. L. , & Cheeseman, I. M. (2016). The molecular basis for centromere identity and function. Nature Reviews Molecular Cell Biology, 17(1), 16–29. 10.1038/nrm.2015.5 PMC8603311 26601620

[cpz170471-bib-0017] Miga, K. H. (2017). The Promises and Challenges of Genomic Studies of Human Centromeres. Progress in Molecular and Subcellular Biology, 56, 285–304. 10.1007/978-3-319-58592-5_12 28840242

[cpz170471-bib-0018] Nagaoka, S. I. , Hassold, T. J. , & Hunt, P. A. (2012). Human aneuploidy: Mechanisms and new insights into an age‐old problem. Nature Reviews Genetics, 13(7), 493–504. 10.1038/nrg3245 PMC3551553 22705668

[cpz170471-bib-0019] Niikura, Y. , & Kitagawa, K. (2016). Immunofluorescence Analysis of Endogenous and Exogenous Centromere‐kinetochore Proteins. Journal of Visualized Experiments, (109), e53732. 10.3791/53732 PMC4828215 26967065

[cpz170471-bib-0020] Ramírez, F. , Dündar, F. , Diehl, S. , Grüning, B. A. , & Manke, T. (2014). deepTools: A flexible platform for exploring deep‐sequencing data. Nucleic Acids Research, 42, (Web Server issue), W187–W191. 10.1093/nar/gku365 PMC4086134 24799436

[cpz170471-bib-0021] Tolomeo, D. , Capozzi, O. , Stanyon, R. R. , Archidiacono, N. , D'Addabbo, P. , Catacchio, C. R. , Purgato, S. , Perini, G. , Schempp, W. , Huddleston, J. , Malig, M. , Eichler, E. E. , & Rocchi, M. (2017). Epigenetic origin of evolutionary novel centromeres. Scientific Reports, 7(1), 41980. 10.1038/srep41980 PMC5290474 28155877

[cpz170471-bib-0022] Trazzi, S. , Perini, G. , Bernardoni, R. , Zoli, M. , Reese, J. C. , Musacchio, A. , & Valle, G. D. (2009). The C‐terminal domain of CENP‐C displays multiple and critical functions for mammalian centromere formation. PloS One, 4(6), e5832. 10.1371/journal.pone.0005832 PMC2688085 19503796

[cpz170471-bib-0023] Yilmaz, D. , Furst, A. , Meaburn, K. , Lezaja, A. , Wen, Y. , Altmeyer, M. , Reina‐San‐Martin, B. , & Soutoglou, E. (2021). Activation of homologous recombination in G1 preserves centromeric integrity. Nature, 600(7890), 748–753. 10.1038/s41586-021-04200-z 34853474

